# Microbiota-Derived Metabolites Suppress Arthritis by Amplifying Aryl-Hydrocarbon Receptor Activation in Regulatory B Cells

**DOI:** 10.1016/j.cmet.2020.03.003

**Published:** 2020-04-07

**Authors:** Elizabeth C. Rosser, Christopher J.M. Piper, Diana E. Matei, Paul A. Blair, André F. Rendeiro, Michael Orford, Dagmar G. Alber, Thomas Krausgruber, Diego Catalan, Nigel Klein, Jessica J. Manson, Ignat Drozdov, Christoph Bock, Lucy R. Wedderburn, Simon Eaton, Claudia Mauri

**Affiliations:** 1 Centre for Adolescent Rheumatology Versus Arthritis at UCL, UCLH and GOSH, London, UK; 2 Centre for Rheumatology Research, Division of Medicine, UCL, London WC1E 6JF, UK; 3 Infection, Immunity and Inflammation Programme, UCL Great Ormond Street Institute of Child Health, London WC1N 1EH, UK; 4 CeMM Research Center for Molecular Medicine of the Austrian Academy of Sciences, Vienna, Austria; 5 Developmental Biology and Cancer Programme, UCL Great Ormond Street Institute of Child Health, London WC1N 1EH, UK; 6 Programa Disciplinario de Inmunología, Facultad de Medicina, Instituto de Ciencias Biomédicas (ICBM), Universidad de Chile, Santiago, Chile; 7 Department of Rheumatology, University College Hospital, London, UK; 8 Bering Limited, London TW2 6EA, UK; 9 NIHR Biomedical Research Centre at Great Ormond Street Hospital, London, UK; 10 Department of Laboratory Medicine, Medical University of Vienna, Vienna, Austria; 11 Max Planck Institute for Informatics, Saarland Informatics Campus, Saarbrücken, Germany

**Keywords:** short chain fatty acid, butyrate, regulatory B cells, autoimmunity, B cells, tryptophan metabolism, aryl-hydrocarbon receptor, rheumatoid arthritis, serotonin, 5-Hydroxyindole-3-acetic acid

## Abstract

The differentiation of IL-10-producing regulatory B cells (Bregs) in response to gut-microbiota-derived signals supports the maintenance of tolerance. However, whether microbiota-derived metabolites can modulate Breg suppressive function remains unknown. Here, we demonstrate that rheumatoid arthritis (RA) patients and arthritic mice have a reduction in microbial-derived short-chain fatty acids (SCFAs) compared to healthy controls and that in mice, supplementation with the SCFA butyrate reduces arthritis severity. Butyrate supplementation suppresses arthritis in a Breg-dependent manner by increasing the level of the serotonin-derived metabolite 5-Hydroxyindole-3-acetic acid (5-HIAA), which activates the aryl-hydrocarbon receptor (AhR), a newly discovered transcriptional marker for Breg function. Thus, butyrate supplementation via AhR activation controls a molecular program that supports Breg function while inhibiting germinal center (GC) B cell and plasmablast differentiation. Our study demonstrates that butyrate supplementation may serve as a viable therapy for the amelioration of systemic autoimmune disorders.

## Highlights

• Stool butyrate levels are reduced in patients with RA compared to healthy controls
• Supplementation with butyrate suppresses arthritis severity in a mouse model
• Suppression of arthritis by butyrate supplementation depends upon AhR^+^Bregs
• Butyrate increases serotonin-derived 5-HIAA, which directly activates AhR^+^Bregs

## Graphical Abstract

**Figure:**
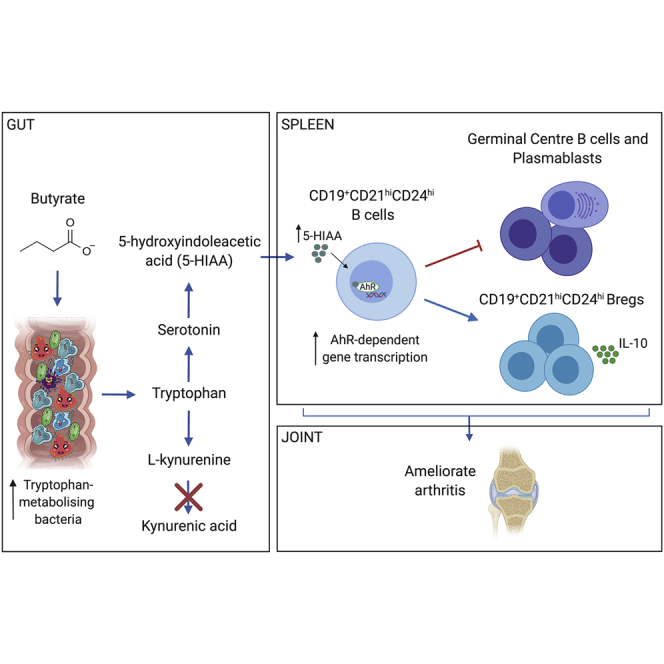

## Teaser

The environmental signals that influence Breg function are not yet fully defined. Here, Rosser et al. demonstrate that the short-chain fatty acid butyrate supports Breg function by increasing the level of the serotonin-derived metabolite 5-Hydroxyindole-3-acetic acid (5-HIAA). 5-HIAA, in turn, activates the aryl-hydrocarbon receptor, a newly discovered transcriptional regulator for Bregs.

## Context and Significance

**Rheumatoid arthritis (RA) is a disease caused by the malfunction of white blood cells. The cause of this malfunction in unclear; however, recent data suggest that changes to the gut bacteria or “microbiota” impact normal immune system function in RA. Researchers at University College London have discovered that supplementation of mice with butyrate, a molecule produced by the microbiota following breakdown of complex dietary starch, suppresses arthritis. Butyrate supplementation acts tosupportthe function of a population of inflammation-suppressing white blood cells called regulatory B cells by changing the composition of microbiota and increasing the production of a serotonin-derived metabolite, 5-Hydroxyindole-3-acetic acid. These data demonstrate that supplementing the diet with certain microbiota-derived molecules may be a promising treatment for arthritis.**

## Introduction

Regulatory B cells (Bregs) are immunosuppressive cells that contribute to the maintenance of immunological tolerance (Mauri and Bosma, 2012). Bregs suppress a variety of immune pathologies including autoimmune diseases through the production of interleukin (IL)-10, IL-35, and transforming growth factor beta 1 (TGFβ1) (Mauri and Bosma, 2012). They inhibit the expansion of pathogenic T cells and other pro-inflammatory lymphocytes, and promote regulatory T cell (Treg) differentiation (Carter et al., 2011, Rosser et al., 2014). Toll-like receptor (TLR) agonists, including lipopolysaccharide (TLR4) and CpG oligo-deoxynucleotides (TLR9), in combination with low grade levels of inflammatory cytokines, for example IFNα and/or IL-1β and IL-6, induce IL-10-producing Breg differentiation (Lampropoulou et al., 2008, Menon et al., 2016, Rosser et al., 2014). The strength of these inflammatory signals is key in determining whether immature B cells develop into Bregs or into mature B cells and antibody-producing plasma cells (Menon et al., 2016). We have recently shown that low-grade inflammatory signals that drive the differentiation of immature B cells into Bregs are provided in the gut-associated lymphoid tissue (GALT) as a result of the interaction between the gut microbiota and the innate immune system (Rosser et al., 2014). Mice depleted of endogenous bacteria following administration of broad-spectrum antibiotics do not develop arthritis or Bregs, suggesting an intricate relationship between microbiota, inflammation, and Breg differentiation (Rosser et al., 2014). The question of whether inflammatory signals produced in response to the microbiota control Breg development alone or whether microbial factors also play a direct role remains unanswered.

Among different gut-microbiota-derived metabolites, the most well-characterized are the end products of dietary fiber fermentation, the short-chain fatty acids (SCFAs). SCFAs serve as an important source of nutrients for intestinal epithelial cells supporting barrier function and act as important cellular mediators regulating gene expression, cell differentiation, and gut tissue development (Nicholson et al., 2012). SCFAs are a potent class of immune-modulatory compounds with the capacity to modulate Treg, T helper 17 (Th17) cells, and macrophage differentiation in the gut and periphery (Arpaia et al., 2013, Chen et al., 2019, Schulthess et al., 2019). In addition to processing dietary-derived material into potentially immunomodulatory compounds, the gut microbiota shapes host responses to xenobiotics, suggesting that transcription factors implicated in xenobiotic metabolism may closely interact and be influenced by the microbial composition and their products (Maurice et al., 2013).

The aryl-hydrocarbon receptor (AhR) is an environmental sensor that binds to a variety of ligands, including xenobiotic ligands such as environmental pollutants (e.g., dioxin) and to physiological compounds derived from the digestion of dietary components by commensal microbiota (Zhou, 2016). AhR plays a pleiotropic role in the maintenance of both the innate and adaptive immune systems in multiple organs and has been shown to be a transcriptional regulator for the development and function of several immune cells including Tregs, Th17, dendritic cells, and more recently, stem cells (Stockinger et al., 2014). In comparison to T cells, we currently lack understanding of how AhR regulates B cell responses. It is known that the levels of AhR expression vary over the lifetime of a B cell and that activation of AhR-dependent gene transcription following ligand binding contributes to the processes controlling B cell differentiation and lineage commitment (Sherr and Monti, 2013). Activation of AhR prevents the differentiation of mature B cells into plasmablasts (Vaidyanathan et al., 2017). Recently, we have shown that AhR is highly expressed in IL-10^+^CD19^+^CD21^hi^CD24^hi^B cells, a subset containing virtually all splenic Bregs, and that AhR promotes and preserves the immunosuppressive state of splenic Bregs by silencing a pro-inflammatory transcriptional program (Piper et al., 2019, Sherr and Monti, 2013). The nature of the ligands that activate AhR and thus control the balance between pro-arthritogenic and regulatory B cell differentiation remains unknown.

Here, we show that supplementation with the SCFA butyrate changes the availability of AhR’s endogenous ligands amplifying AhR-dependent gene transcription in CD19^+^CD21^hi^CD24^hi^B cells. Butyrate supplementation augments AhR ligand availability by supporting the growth of tryptophan bacteria that influence the metabolism of tryptophan, which increases the production of the main metabolite of serotonin, 5-hydroxyindole-3-acetic acid (5-HIAA). 5-HIAA, in turn, activates AhR-dependent gene transcription in B cells supporting Breg function and inhibiting germinal center (GC) B cell and plasma cell differentiation. This results in an amelioration of arthritis.

## Results

### Rheumatoid Arthritis (RA) Patients Have Lowered Stool Butyrate, Which Correlates with a Reduction in CD19^+^CD24^hi^CD38^hi^B Cell and IL-10^+^Breg Frequency

**Figure 1:**
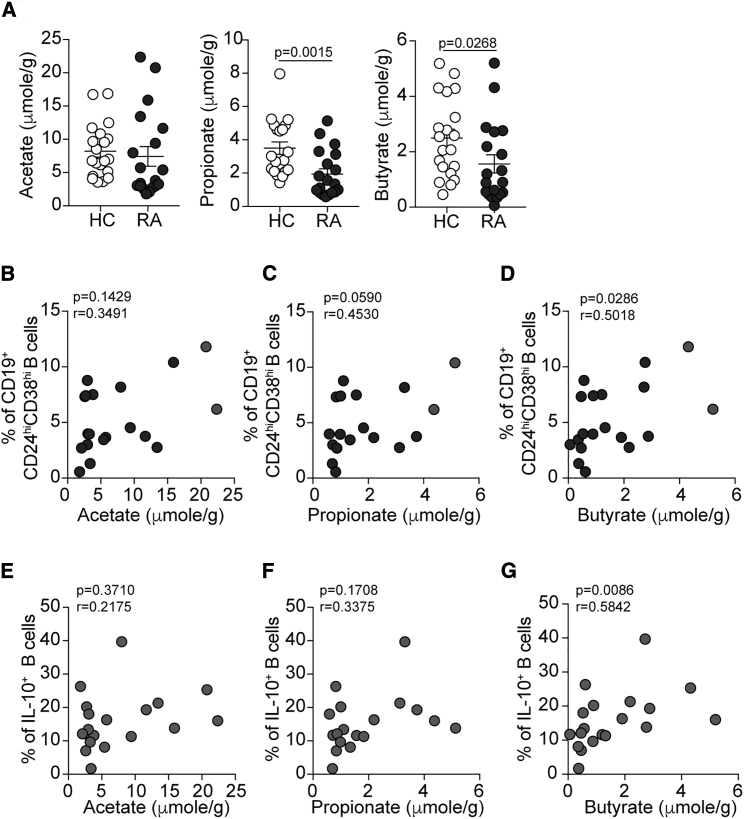
Butyrate Levels Correlate with Immature B Cells in Patients with Inactive Rheumatoid Arthritis (A) Representative histograms show stool acetate, propionate, and butyrate levels in healthy controls (HC, n = 20) and RA patients (n = 19) as measured by high-performance liquid chromatography. (B–D) Scatterplots show correlation between stool (B) acetate, (C) propionate, and (D) butyrate levels and CD19^+^CD24^hi^CD38^hi^B cell frequency in the peripheral blood of RA patients (n = 19). (E–G) Scatterplots show correlation between stool (E) acetate, (F) propionate, and (G**)** butyrate levels and IL-10^+^B cell frequency in the peripheral blood of RA patients (n = 19). Data represent mean ± SE (A, Mann-Whitney test; B–G, Spearmen correlation). See also Figure S1.

Recent research has established that dysbiosis of the gut microbiota may be a contributing factor to RA pathogenesis (Scher et al., 2013, Zhang et al., 2015). How dysbiosis contributes to abnormal immune cell function in human arthritic disease remains unknown. We have previously reported that the frequency of CD19^+^CD24^hi^CD38^hi^B cells, which contain the highest proportion of IL-10^+^Bregs, is inversely correlated with clinical disease severity in RA (Blair et al., 2010, Flores-Borja et al., 2013). Based on evidence that microbiota-derived SCFAs are essential for the maintenance of immunological homeostasis, we hypothesized that dysbiosis in RA could affect SCFAs, resulting in abnormal B cell homeostasis and a reduction in Breg frequency. We collected stool and paired serum samples from RA patients, and SCFA levels were compared to age- and sex-matched healthy controls (HCs) (patient demographics are reported in Table S1). Due to the limitations imposed by the low number of Bregs present in RA patients with active disease (Flores-Borja et al., 2013), only inactive patients were recruited to this study. We identified that there was a significant reduction in butyrate and propionate, and no difference in acetate, in stool samples of RA patients compared to HCs (Figure 1A). In serum samples, there was no difference in propionate or butyrate but a significant increase in acetate levels in RA patients compared to HCs (Figure S1A). As previously reported, we also found a trend for a negative correlation between age and butyrate or acetate levels in HCs but not RA patients (Figure S1B) (Nagpal et al., 2018).

To investigate whether changes in SCFA levels were associated with abnormal B cell and Breg homeostasis, peripheral blood B cell subset and IL-10^+^B cell frequency were enumerated in paired blood samples from RA patients and correlated with stool acetate, propionate, and butyrate levels. In RA patients, we reported a significant positive correlation between butyrate levels and the frequency of total CD19^+^CD24^hi^CD38^hi^B cells and with IL-10^+^B cells (Figures 1D and 1G). There was no significant correlation between propionate or acetate and CD19^+^CD24^hi^CD38^hi^B cells or IL-10^+^Breg frequency (Figures 1B–1F). There was no significant correlation between stool SCFAs and CD19^+^CD24^hi^CD38^int^ (mature naive) or CD19^+^CD24^hi^CD38^neg^ (memory) B cell frequency (Figures S1C and S1D). Despite these data only being correlative, they may suggest a role for butyrate in supporting Breg homeostasis in arthritic disease.

### Butyrate Supplementation Suppression of Experimental Arthritis Is Breg Dependent

**Figure 2:**
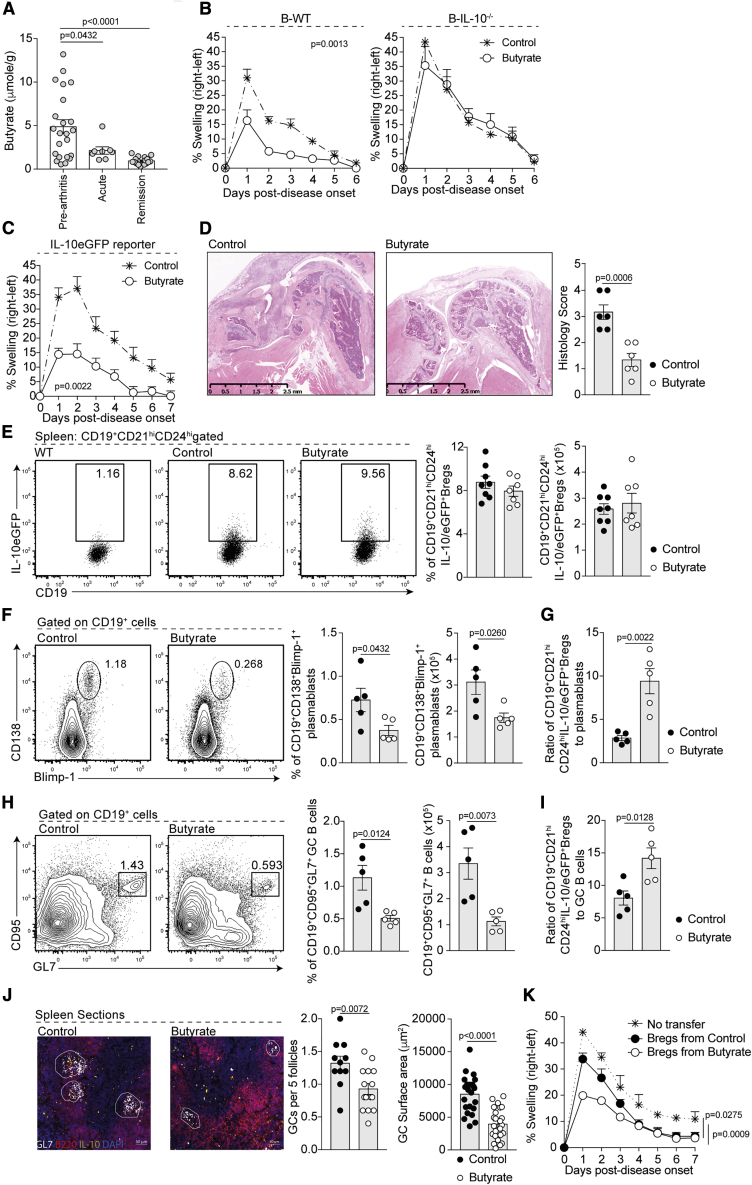
Butyrate Supplementation Suppresses Arthritis by Skewing the B Cell Compartment in Favor of a Regulatory Phenotype (A) Stool butyrate levels in WT mice pre-arthritis (n = 23), with acute arthritis (n = 8), and in remission from arthritis (n = 18) as measured by high-performance liquid chromatography (cumulative data are shown). (B) Mean clinical score of control (cumulative n = 25) and butyrate-supplemented B-WT chimeric mice or B-IL-10^−/−^chimeric mice (n = 8 per group) (one representative experiment of two experiments is shown); y axis shows percentage swelling in antigen-injected knee compared to control knee. (C) Mean clinical score of control (cumulative n = 15) and butyrate-supplemented IL-10eGFP reporter mice (cumulative n = 13); y axis shows percentage swelling in antigen-injected knee compared to control knee (one representative experiment of two experiments is shown). (D) Representative H&E staining of knee joints from control and butyrate-supplemented IL-10eGFP reporter mice (left) and blinded histology scores (right) of joint damage. (E) Representative flow cytometry plots (left) and bar charts (right) showing CD19^+^CD21^hi^CD24^hi^IL-10eGFP^+^Breg frequency and number in control (cumulative n = 15) and butyrate-supplemented mice (cumulative n = 13) (one representative experiment of three experiments is shown). (F) Representative flow cytometry plots (left) and bar charts (right) showing CD19^+^CD138^+^Blimp-1^+^plasmablast frequency and number in control and butyrate-supplemented mice (cumulative n = 11 per group, one representative experiment of two experiments is shown). (G) Bar charts show ratio of CD19^+^CD21^hi^CD24^hi^IL-10eGFP^+^Bregs to plasmablast in control and butyrate-supplemented mice (cumulative n = 11 per group, one representative experiment of two experiments is shown). (H) Representative flow cytometry plots (left) and bar chart (right) shows the percentage and number of CD19^+^CD95^+^GL7^+^ germinal center (GC) B cells in control and butyrate-supplemented mice (cumulative n = 11 per group, one representative experiment of three experiments is shown). (I) Bar chart shows ratio of CD19^+^CD21^hi^CD24^hi^IL-10eGFP^+^Bregs to GC B cells in control and butyrate-supplemented mice (cumulative n = 11, one representative experiment of two experiments is shown). (J) Representative immunofluorescence blinded histological analysis of the number and size of GC control and butyrate-supplemented mice (original magnification 20×, n = 3). (K) Mean clinical score following transfer of CD19^+^CD21^hi^CD24^hi^IL-10eGFP^+^Bregs from control (cumulative n = 6) or butyrate-supplemented mice (cumulative n = 6), a control group that did not receive a transfer; y axis shows percentage swelling in antigen-injected knee compared to control knee (cumulative n = 8) (one representative experiment of two experiments is shown). Cells were isolated at day 7 post-disease onset. Data represent mean ± SE (A, one-way ANOVA; B, C, and K, two-way ANOVA; D–J, Student’s t test). See also Figures S2–S4.

To elucidate the mechanisms by which butyrate may influence the Breg compartment, we utilized the antigen-induced model of arthritis (AIA). In this model, both Breg function and arthritis severity are dependent upon the gut microbiota (Rosser et al., 2014). Analysis of SCFA levels in the stool of arthritic mice revealed a reduction in butyrate and acetate during the acute and remission phase of arthritis compared to pre-arthritic mice (Figures 2A and S2A). Propionate was significantly reduced during the remission phase of disease, but there were no differences in the acute phase of disease compared to pre-arthritic mice (Figure S2A). These results suggest that the observed defect in SCFA production in arthritic mice, once established, cannot be reversed in spite of the reduced inflammation observed during disease remission. In line with reduced SCFAs, the bacterial families *Lactobacillaceae*, *Rikenellaceae*, and *Bacteroidaceae* were reduced in the stool of arthritic mice compared to naive mice (Figure S2B). Members of these bacterial families form a common functional group of bacteria that metabolize non-digestible carbohydrates into the immunogenic SCFA (Basson et al., 2016). Conversely, we detected an increase in *Desulfovibrionaceae*, *Deferribacteraceae*, *Sutterellaceae*, and *Prevotellaceae* families in the stool of arthritic versus naive mice (Figure S2B).

Previously published research has demonstrated that supplementation with SCFAs, and in particular butyrate, has an immunosuppressive effect in diseases including diabetes and colitis (Mariño et al., 2017, Smith et al., 2013). To evaluate the contribution of each individual SCFA in controlling the severity of arthritis and to determine the possible role of B cells in mediating suppression, acetate, propionate, and butyrate were supplemented in the drinking water of wild-type (WT) mice and B-cell-deficient (μMT) mice prior to disease induction. Control mice for both genotypes received drinking water that was salt and pH balanced (hereafter referred to as the control group). Only supplementation with butyrate, but not acetate and propionate, reduced arthritis in WT mice compared to control mice (Figures 2B, S2C, and S2D). Butyrate supplementation failed to suppress disease in B-cell-deficient mice (μMT) (Figure S2C), demonstrating that under these experimental conditions, B cells are key in mediating the beneficial effects of butyrate supplementation. As seen in WT mice, supplementation with acetate or propionate did not affect disease severity in μMT mice (Figures S2D and S2E). Furthermore, butyrate supplementation failed to suppress disease in mixed bone marrow chimeric mice lacking IL-10-producing B cells (Figure 2B), pinpointing the requirement of Bregs in the butyrate-mediated suppression of arthritis. Butyrate suppressed the severity of collagen-induced arthritis, further strengthening the anti-inflammatory role of butyrate in arthritic disease (Figure S2F).

### Butyrate Supplementation Skews the B Cell Compartment in Favor of a Regulatory Phenotype

To investigate the effect of butyrate supplementation on both pro-arthritogenic cells and Bregs, we next took advantage of IL-10eGFP reporter mice, allowing the visualization of B cells actively transcribing IL-10 (Madan et al., 2009). Amelioration of disease in butyrate-supplemented IL-10eGFP reporter mice was similar to WT mice (Figures 2C and 2D respectively showing clinical score and histological changes of the joints). There was no increase in serum butyrate levels in butyrate-supplemented versus control mice (Figure S2G). Suppression of disease by butyrate supplementation was associated with a reduction in TNFα, IL-6, and MCP-1 (CCL2) production by total lymphocytes isolated from the draining lymph node (LN) (Figure S3A) and by a decrease in IL-1β^+^CD11b^+^ and IL-6^+^CD11b^−^ splenocyte frequency compared to control mice (Figures S3B and S3C). Analysis of the T cell compartment revealed a reduction in CD4^+^IL-17^+^T cell frequency and IL-17 production by total lymphocytes in the draining LN of butyrate-supplemented versus control mice (Figures S3D and S3E). Butyrate supplementation did not affect IFNγ production or CD4^+^IFNγ^+^T cell frequency, nor Foxp3^+^Treg frequency or Foxp3^+^Treg number (Figures S3D–S3F). However, butyrate-mediated suppression was reduced in mice when Tregs were depleted following anti-CD25 depleting antibody treatment (Figures S3G and S3H). Therefore, Tregs also play a role in mediating the suppression of arthritis by butyrate supplementation, supporting data in the literature showing the pleiotropic immunomodulatory effect of this SFCA (Corrêa-Oliveira et al., 2016).

We next assessed the effect of butyrate supplementation on the differentiation of Bregs, identified here as IL-10eGFP^+^CD19^+^CD21^hi^CD24^hi^B cells (Piper et al., 2019). We found that IL-10eGFP^+^CD19^+^CD21^hi^CD24^hi^Breg number and frequency were similar between butyrate-supplemented and control mice (Figure 2E). The frequency and total number of IL-10eGFP^+^CD5^+^B cells (O’Garra et al., 1992), Tim-1^+^B cells (Ding et al., 2011), and CD5^+^CD1d^+^B10 cells (Yanaba et al., 2008), all of which have previously been ascribed with regulatory capacity, also remained unchanged following butyrate supplementation (Figures S4A–S4F). There was no difference in the frequency and/or number of splenic follicular (FO) B cells, transitional-1 (T1) B cells, or total CD19^+^CD21^hi^CD24^hi^B cells (Figures S4G–S4J). However, there was a significant reduction of CD19^+^CD138^+^Blimp-1^+^plasmablast and CD19^+^CD95^+^GL7^+^GC B cell frequency and number between butyrate-supplemented and control mice (Figures 2F and 2H). Blinded histological analyses further confirmed a reduction in the number of GCs per B cell follicle and in the size of GCs in the spleens of butyrate-supplemented versus control mice (Figure 2J). Thus, butyrate supplementation had increased the ratio of IL-10eGFP^+^CD19^+^CD21^hi^CD24^hi^Bregs to plasmablasts and IL-10eGFP^+^CD19^+^CD21^hi^CD24^hi^Bregs to GC B cells compared to control mice (Figures 2G and 2I).

To determine whether butyrate supplementation affects the immunosuppressive function of Bregs, an equal number of IL-10eGFP^+^CD19^+^CD21^hi^CD24^hi^Bregs was isolated from butyrate-supplemented or control IL-10eGFP reporter mice and transferred into syngeneic arthritic hosts. IL-10eGFP^+^CD19^+^CD21^hi^CD24^hi^Bregs from butyrate-supplemented mice displayed enhanced suppressive capacity upon adoptive transfer compared to IL-10eGFP^+^CD19^+^CD21^hi^CD24^hi^Bregs from control mice (Figure 2K). These results demonstrated that butyrate supplementation concurrently increases Breg suppressive capacity and limits GC B cell and plasmablast differentiation.

### Suppression of Disease by Butyrate Supplementation Requires B Cell Expression of AhR

**Figure 3:**
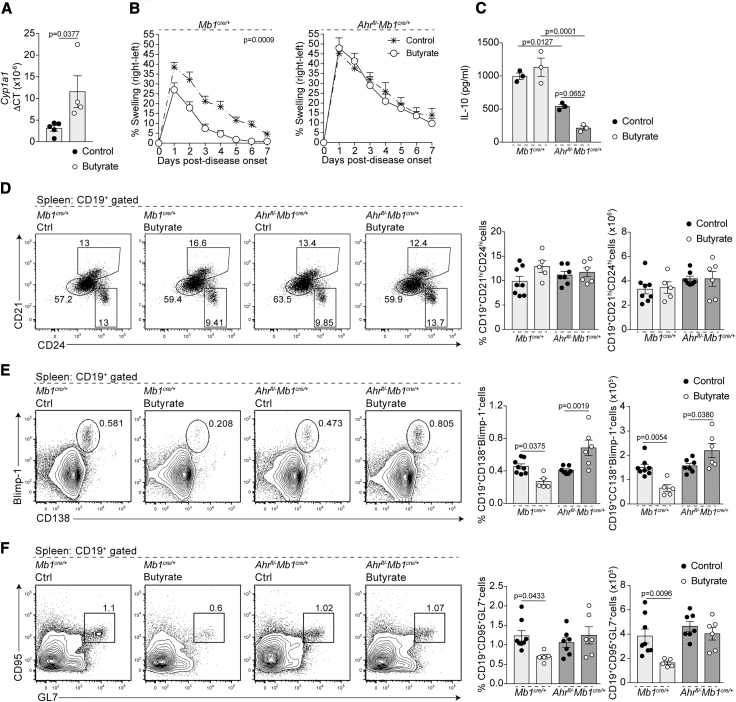
Suppression of Arthritis by Butyrate Supplementation Depends upon AhR Activation and IL-10 Expression in B Cells (A) Bar chart shows expression of *Cyp1a1* relative to *β-actin* in splenic B cells isolated from control or butyrate-supplemented mice (cumulative n = 5, one representative experiment of two experiments is shown). (B) Mean clinical score of control and butyrate-supplemented *Mb1*^*cre/+*^ mice or *Ahr*^*fl/−*^*Mb1*^*cre/+*^ mice; y axis shows percentage swelling in antigen-injected knee compared to control knee (cumulative n = 15 per group, one representative experiment of five experiments is shown). (C) IL-10 production by splenic B cells isolated from control *Mb1*^*cre/+*^ mice, butyrate-supplemented *Mb1*^*cre/+*^ mice, control *Ahr*^*fl/−*^*Mb1*^*cre/+*^ mice, and butyrate-supplemented *Ahr*^*fl/−*^*Mb1*^*cre/+*^ mice at day 7 post-disease onset as measured by ELISA (cumulative n = 3 per group). (D) Representative flow cytometry plots and bar charts showing the frequency and number of CD19^+^CD21^hi^CD24^hi^B cells in control *Mb1*^*cre/+*^ mice (cumulative n = 8), butyrate-supplemented *Mb1*^*cre/+*^ mice (cumulative n = 5), control *Ahr*^*fl/−*^*Mb1*^*cre/+*^ mice (n = 7), and butyrate-supplemented *Ahr*^*fl/−*^*Mb1*^*cre/+*^ mice (cumulative n = 6) at day 7 post-disease onset (cumulative data are shown). (E) Representative flow cytometry plots and bar charts showing the frequency and number of CD19^+^CD138^+^Blimp-1^+^B cells in control *Mb1*^*cre/+*^ mice (cumulative n = 8), butyrate-supplemented *Mb1*^*cre/+*^ mice (cumulative n = 5), control *Ahr*^*fl/−*^*Mb1*^*cre/+*^ mice (cumulative n = 7), and butyrate-supplemented *Ahr*^*fl/−*^*Mb1*^*cre/+*^ mice (cumulative n = 6) (cumulative data are shown). (F) Representative flow cytometry plots and bar charts showing the frequency and number of CD19^+^CD95^+^GL7^+^B cells in control *Mb1*^*cre/+*^ mice (cumulative n = 8), butyrate-supplemented *Mb1*^*cre/+*^ mice (cumulative n = 5), control *Ahr*^*fl/−*^*Mb1*^*cre/+*^ mice (cumulative n = 7), and butyrate-supplemented *Ahr*^*fl/−*^*Mb1*^*cre/+*^ mice (cumulative n = 6) (cumulative data are shown). Cells were isolated at day 7 post-disease onset. Data represent mean ± SE (A, Student’s t test; C, E, and F, one-way ANOVA; B, two-way ANOVA). See also Figures S5 and S6.

We have recently demonstrated that there is higher expression of the AhR in IL-10eGFP^+^CD19^+^CD21^hi^CD24^hi^Bregs and that activation of AhR, which can be used as a proxy for *Il10* transcription, contributes to the induction of a transcriptional program that controls IL-10eGFP^+^CD19^+^CD21^hi^CD24^hi^Breg suppressive function (Piper et al., 2019). This, taken together with previous findings demonstrating that AhR suppresses plasmablast differentiation (Vaidyanathan et al., 2017), led us to hypothesize that butyrate supplementation suppresses arthritis and alters B cell subset composition either directly or indirectly by activation of AhR in B cells. In line with this hypothesis, the expression of *Cyp1a1*, a prototypical reporter gene of AhR activation, was significantly upregulated in B cells isolated from butyrate-supplemented mice compared to control mice (Figure 3A). To confirm a role for AhR in the immune-modulatory effect of butyrate supplementation on the B cell compartment and arthritis severity, we took advantage of *Ahr*^*fl/−*^*Mb1*^*cre/+*^ mice, which have a B cell specific deletion of AhR (Villa et al., 2017). Similarly to WT mice, butyrate supplementation suppressed arthritis severity and CD4^+^IL-17^+^T cell frequency only in AhR-sufficient *Mb1*^*cre/+*^ mice but not in *Ahr*^*fl/−*^*Mb1*^*cre/+*^ mice (Figures 3B and S5A). As recently shown, B cells from *Ahr*^*fl/−*^*Mb1*^*cre/+*^ mice released less IL-10 compared to those isolated from *Mb1*^*cre/+*^ mice; a reduction that was not restored following butyrate supplementation (Piper et al., 2019) (Figure 3C). Butyrate supplementation did not alter CD19^+^CD21^hi^CD24^hi^B cell frequency and number in *Mb1*^*cre/+*^ or *Ahr*^*fl/−*^*Mb1*^*cre/+*^ mice compared to control groups (Figure 3D). Corroborating the results in Figures 2F and 2H, butyrate supplementation reduced CD19^+^CD138^+^Blimp-1^+^plasmablast and CD19^+^CD95^+^GL7^+^GC B cell frequency and number in *Mb1*^*cre/+*^ mice but failed to suppress CD19^+^CD138^+^Blimp-1^+^plasmablast and CD19^+^CD95^+^GL7^+^GC B cell frequency and number in *Ahr*^*fl/−*^*Mb1*^*cre/+*^ mice (Figures 3E and 3F). Although Treg frequency and number were unaffected by butyrate supplementation (Figures S5B and S5C), CD3^+^CD4^+^CD25^+^Tregs isolated from butyrate-supplemented *Mb1*^*cre/+*^ mice displayed enhanced suppressive capacity upon adoptive transfer into WT mice (Figure S5D). In contrast, Tregs isolated from both control and butyrate-supplemented *Ahr*^*fl/−*^*Mb1*^*cre/+*^ mice failed to suppress disease on adoptive transfer (Figure S5D).

As inflammation is a driver of Breg differentiation and function, and because *Ahr*^*fl/−*^*Mb1*^*cre/+*^ mice develop an exacerbated arthritic inflammation compared to *Mb1*^*cre/+*^ mice, we next tested the effect of butyrate supplementation in chimeric mice reconstituted with a 1:1 mix of bone marrow cells from CD45.1 WT and CD45.2 AhR^−/−^ mice (Figure S6A). Under these conditions, WT and AhR^−/−^ B cells are exposed to identical inflammatory signals following arthritis induction. Confirming the results in Figure 3, the frequency and number of WT CD45.1^+^CD45.2^−^IL-10^+^CD19^+^CD21^hi^CD24^hi^B cells was unaffected by butyrate supplementation, whereas AhR^−/−^CD45.1^−^CD45.2^+^CD19^+^CD21^hi^CD24^hi^B cells failed to differentiate into IL-10^+^CD19^+^CD21^hi^CD24^hi^Bregs in both control and butyrate-supplemented mice (Figure S6B). In addition, butyrate supplementation reduced the frequency and number of plasmablasts and GC B cells within CD45.1 WT-derived cells but not in CD45.2 AhR^−/−^-derived cells (Figures S6C and S6D).

### Butyrate Supplementation Supports Breg Suppressive Function and Controls B Cell Differentiation Partly via an AhR-Dependent Transcriptional Program

**Figure 4:**
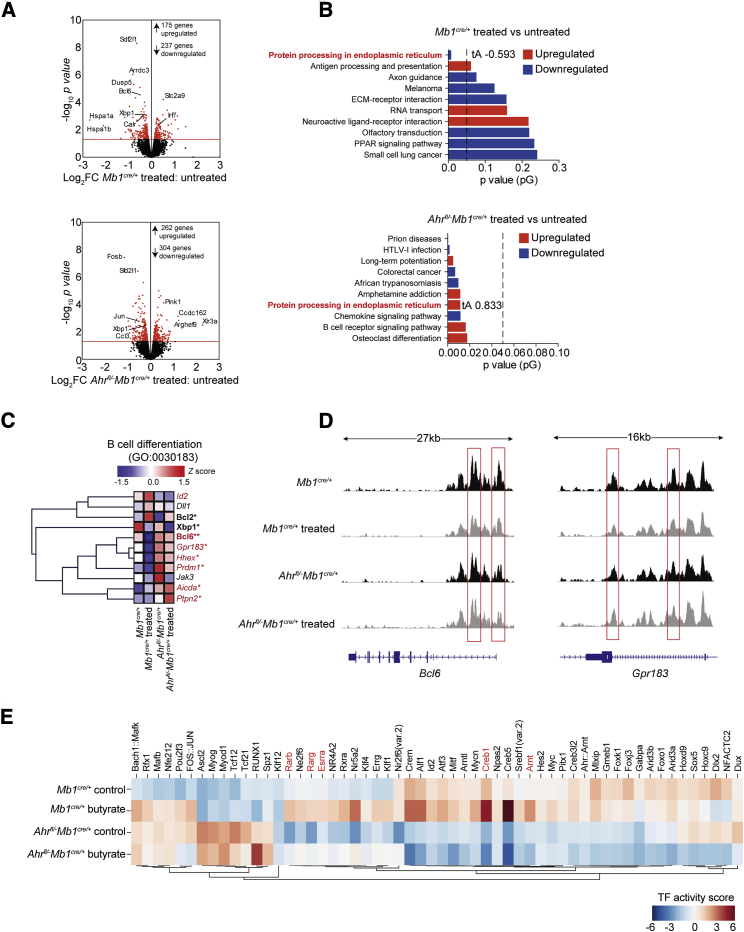
Butyrate Supplementation Modulates the Transcriptional and Epigenetic Landscape of CD19^+^CD21^hi^CD24^hi^B Cells in an AhR-Dependent Manner (A) Volcano plots shows log_2_ fold change (FC) in gene expression between CD19^+^CD21^hi^CD24^hi^B cells isolated from butyrate-supplemented *Mb1*^*cre/+*^ mice compared to control *Mb1*^*cre/+*^ mice (top plot) and between butyrate supplemented *Ahr*^*fl/−*^*Mb1*^*cre/+*^ compared to control *Ahr*^*fl/−*^*Mb1*^*cre/+*^ mice (bottom plot). Red dots represent significant DEGs, with the red line denoting a cut off p value of <0.05. (B) Signaling pathway impact analysis (SPIA) ranked on significance (pG) comparing the over-represented (red) and under-represented (blue) pathways in butyrate-supplemented compared to control CD19^+^CD21^hi^CD24^hi^B cells from *Mb1*^*cre/+*^ mice (top graph) and *Ahr*^*fl/−*^*Mb1*^*cre/+*^ mice (bottom graph). The total perturbation accumulation (tA) score is listed for the “protein processing in endoplasmic reticulum” pathway. (C) Heatmap shows the expression of B cell differentiation genes in CD19^+^CD21^hi^CD24^hi^B cells isolated from control *Mb1*^*cre/+*^ mice, butyrate-supplemented *Mb1*^*cre/+*^ mice, control *Ahr*^*fl/−*^*Mb1*^*cre/+*^ mice, and butyrate-supplemented *Ahr*^*fl/−*^*Mb1*^*cre/+*^ mice. Mean z scores were calculated from log CPM values. Samples highlighted in red are significantly differentially expressed between CD19^+^CD21^hi^CD24^hi^B cells isolated from butyrate-supplemented *Mb1*^*cre/+*^ mice compared to butyrate-supplemented *Ahr*^*fl/−*^*Mb1*^*cre/+*^ mice. Samples highlighted in bold are significantly differentially expressed between CD19^+^CD21^hi^CD24^hi^B cells isolated from butyrate-supplemented *Mb1*^*cre/+*^ mice compared to control *Mb1*^*cre/+*^ mice. (D) Representative ATAC-seq tracks for the *Bcl6* and *Gpr183* loci in CD19^+^CD21^hi^CD24^hi^B cells from butyrate-supplemented or control *Mb1*^*cre/+*^ and *Ahr*^*fl/−*^*Mb1*^*cre/+*^ mice (n = 3). Track heights between samples are normalized through group autoscaling. For RNA-seq data, n = 3 per condition and genotype. (E) Heatmap shows inferred transcription factor activity scores based on accessibility at transcription factor binding motifs in CD19^+^CD21^hi^CD24^hi^B cells isolated from control *Mb1*^*cre/+*^ mice, butyrate-supplemented *Mb1*^*cre/+*^ mice, control *Ahr*^*fl/−*^*Mb1*^*cre/+*^ mice, and butyrate-supplemented *Ahr*^*fl/−*^*Mb1*^*cre/+*^ mice as measured by ATAC-seq. AhR co-factors are highlighted in red. For ATAC-seq data, n = 3 for *Mb1*^*cre/+*^ mice and n = 2 for *Ahr*^*fl/−*^*Mb1*^*cre/+*^ mice. For RNA-seq data, n = 3 per group. Cells were isolated at day 7 post-disease onset. See also Figure S7.

To understand how butyrate supports Breg suppressive function and suppresses GC B cell and plasmablast differentiation, we compared the gene expression profiles and chromatin accessibility of CD19^*+*^CD21^hi^CD24^hi^B cells isolated from butyrate-supplemented and control *Mb1*^*cre/+*^ *and Ahr*^*fl/−*^*Mb1*^*cre/+*^mice. This population was chosen as we have shown that differentiation of CD19^+^CD21^hi^CD24^hi^B cells into IL-10^+^Bregs is dependent upon AhR (Piper et al., 2019) (Figure S6). There were 412 significantly differentially expressed genes (DEGs) between control and butyrate-supplemented *Mb1*^*cre/+*^CD19^*+*^CD21^hi^CD24^hi^B cells (Figure 4A). There were more changes (566 significantly DEGs) in butyrate-supplemented versus control *Ahr*^*fl/−*^*Mb1*^*cre/+*^CD19^*+*^CD21^hi^CD24^hi^B cells. This suggests that, as well as being necessary to mediate some of butyrate’s effects on gene expression, AhR also represses the expression of a number of genes that would otherwise be altered by butyrate treatment (Figure 4A). Signaling pathway impact analysis (SPIA) revealed that the “protein processing in the endoplasmic reticulum” pathway, previously associated with the differentiation of B cells into plasma cells (Iwakoshi et al., 2003), was significantly downregulated by butyrate supplementation in *Mb1*^*cre/+*^CD19^*+*^CD21^hi^CD24^hi^B cells and significantly upregulated *Ahr*^*fl/−*^*Mb1*^*cre/+*^CD19^+^CD21^hi^CD24^hi^B cells (Figure 4B). Based on this observation, we interrogated DEGs in the B cell differentiation Gene Ontology term (GO:0030183) and compared the effect of butyrate supplementation on gene expression in both genotypes. B cell lymphoma 6 protein (*Bcl6*), a master regulator of GC B cell differentiation, and the orphan G protein-coupled receptor (*Gpr183*), important in extra-follicular plasmablast differentiation (Gatto et al., 2009), were among the genes reduced in CD19^+^CD21^hi^CD24^hi^B cells from *Mb1*^*cre/+*^ mice compared to *Ahr*^*fl/−*^*Mb1*^*cre/+*^ mice after butyrate supplementation (Figure 4C). Conversely, the expression of *Id2*, a negative regulator of B cell maturation (Becker-Herman et al., 2002), was upregulated in CD19^+^CD21^hi^CD24^hi^B cells from *Mb1*^*cre/+*^ mice, but not from *Ahr*^*fl/−*^*Mb1*^*cre/+*^ mice after butyrate supplementation (Figure 4C). To investigate whether there was an AhR-independent mechanism in the Breg-mediated regulation of arthritis by butyrate supplementation, we performed a four-way comparison analysis among all the groups (Figure S7A). This analysis also highlights the baseline transcriptional changes between control *Mb1*^*cre/+*^ versus *Ahr*^*fl/−*^*Mb1*^*cre/+*^ mice, which we have defined in a previously published manuscript (Piper et al., 2019). We found that 71 significantly DEGs were regulated in both *Mb1*^*cre/+*^ versus *Ahr*^*fl/−*^*Mb1*^*cre/+*^ mice by butyrate supplementation (Figure S7A; Table S2). There were 195 significantly DEGs observed only in *Mb1*^*cre/+*^ mice but not *Ahr*^*fl/−*^*Mb1*^*cre/+*^ mice following butyrate supplementation after genotype confounding genes had been removed; the majority of these genes were structural proteins (Figure S7A; Table S3).

Similarly to the baseline transcriptional changes between *Mb1*^*cre/+*^ versus *Ahr*^*fl/−*^*Mb1*^*cre/+*^ mice observed during the transcriptome analysis, there were clear differences in chromatin accessibility as measured by assay for transposase-accessible chromatin using sequencing (ATAC-seq) between control *Mb1*^*cre/+*^ mice and *Ahr*^*fl/−*^*Mb1*^*cre/+*^ mice (Figure S7B). However, corroborating the results in Figure 4C, there was decreased accessibility in several B cell maturation genes, including the *Bcl6* and *Gpr183* loci, upon butyrate supplementation exclusively in *Mb1*^*cre/+*^CD19^*+*^CD21^hi^CD24^hi^B cells (Figure 4D). ATAC-seq analysis also revealed that butyrate supplementation did not alter accessibility of the AhR:ARNT specific binding motifs (Abel and Haarmann-Stemmann, 2010), but did increase accessibility at binding motifs for transcription factors that have been identified to function alongside the AHR:ARNT heterodimer, including *Esrra* (estrogen receptor alpha), *Creb1*, and *Rarb/Rarg* (Retinoic acid receptor) (Figure 4E) (Jackson et al., 2015). We confirmed that, similarly to Tregs and monocytes (Arpaia et al., 2013, Schulthess et al., 2019), butyrate acted as a histone deacetylase inhibitor (HDACi) on splenic B cells *in vitro*, providing a partial explanation of its effect on the transcriptional and epigenetic landscape of CD19^+^CD21^hi^CD24^hi^B cells (Figure S7C).

**Figure 5:**
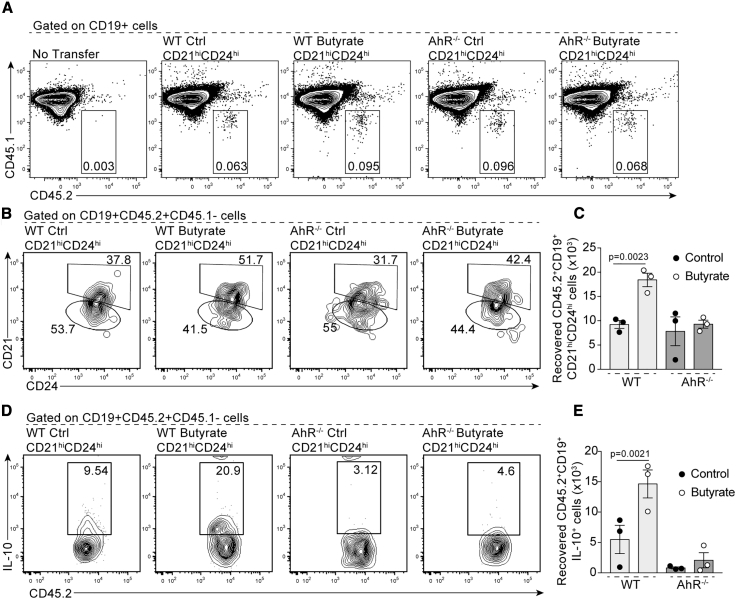
CD45.2^+^CD19^+^CD21^hi^CD24^hi^B Cells from Butyrate-Supplemented WT but Not AhR^−/−^ Mice Retain Their Phenotype and Differentiate in IL-10^+^Bregs upon Adoptive Transfer (A and B) Representative flow cytometry plots show (A) CD45.2^+^CD19^+^B cell and (B) CD45.2^+^CD19^+^CD21^hi^CD24^hi^ B cell frequency in CD45.1 congenic WT mice that had received a transfer of CD19^+^CD21^hi^CD24^hi^B cells isolated from control or butyrate-supplemented WT or AhR^−/−^ mice. (C) Bar chart shows number of CD45.2^+^CD19^+^CD21^hi^CD24^hi^B cells recovered post-transfer from CD45.1 congenic WT mice that had received a transfer of CD19^+^CD21^hi^CD24^hi^B cells isolated from control or butyrate-supplemented WT or AhR^−/−^ mice. (D) Representative flow cytometry plots and bar charts show CD45.2^+^CD19^+^IL-10^+^B cell frequency in CD45.1 congenic WT mice that had received a transfer of CD19^+^CD21^hi^CD24^hi^B cells isolated from control or butyrate-supplemented WT or AhR^−/−^ mice. (E) Bar chart shows number of CD45.2^+^CD19^+^IL-10^+^B cells recovered post-transfer from CD45.1 congenic WT mice that had received a transfer of control or butyrate-supplemented WT or AhR^−/−^ mice. Cells were isolated at 48 h post-transfer (cumulative n = 3 per group, cumulative data are shown). Data represent mean ± SE (C and E, one-way ANOVA).

To investigate whether changes in the epigenetic and transcriptional profile of AhR^+^CD19^+^CD21^hi^CD24^hi^B cells and AhR^−^CD19^+^CD21^hi^CD24^hi^B cells following butyrate supplementation had altered their stability and ability to differentiate into IL-10 competent Bregs, we followed the fate of adoptively transferred CD19^+^CD21^hi^CD24^hi^B cells isolated from butyrate-supplemented and control WT or global AhR^−/−^ in congenic CD45.1 recipient WT mice. A higher number of donor CD45.2^+^CD19^+^CD21^hi^CD24^hi^B cells were recovered post-transfer, and more transferred cells were IL-10^+^ when cells were isolated from butyrate-supplemented WT mice compared to control WT mice (Figures 5A–5D). The rate of cell recovery was not altered by butyrate supplementation when cells were isolated from AhR^−/−^mice and there was a failure of CD45.2^+^CD19^+^CD21^hi^CD24^hi^B cells to differentiate into IL-10^+^Bregs (Figures 5A–5D).

### Butyrate Changes the Levels of Availability of Microbiota-Induced AhR Ligands

**Figure 6:**
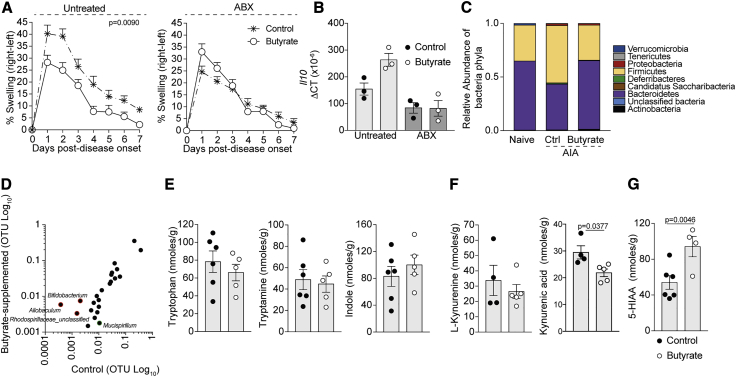
Butyrate Supplementation Increases the Availability of AhR Ligands (A) Mean clinical score of control and butyrate-supplemented ABX-treated or untreated mice; y axis shows percentage swelling in antigen-injected knee compared to control knee (cumulative n = 8 per group, one representative experiment of two experiments is shown). (B) Bar chart shows expression of *Il10* relative to *β-actin* in splenic B cells isolated from ABX-treated WT or untreated mice (cumulative n = 3 per group). (C) Bar chart shows relative abundance of bacterial phyla in the stool of naive, control arthritic, or butyrate-supplemented arthritic mice (n = 4 per group). (D) XY graph shows operational taxonomic units (OTUs) of bacterial genera in butyrate-supplemented and control arthritic mice (n = 4 per group). (E–G) Bar charts shows levels of tryptophan, tryptamine, indole (E), L-Kynurenine, Kynurenic Acid (KYNA) (F), and 5-HIAA (G) in the stool of control arthritic WT and butyrate-supplemented arthritic mice (cumulative n = 5 per group). Data represent mean ± SE (A, two-way ANOVA; B, one-way ANOVA; E–G, Student’s t test). See also Figure S7.

The microbiota is an important contributor to the pool of endogenous AhR ligands, and we and others have previously shown that changes in the composition of the gut microbiota alters the differentiation of CD19^+^CD21^hi^CD24^hi^B cells into functionally suppressive Bregs (Alhabbab et al., 2015, Rosser et al., 2014). Having excluded a direct effect for butyrate in activating AhR, as butyrate did not upregulate the marker of AhR-activation *Cyp1a1* compared to vehicle-treated B cells *in vitro* (Figure S7D), we investigated whether the endogenous microbiota or their metabolites are important in the butyrate-mediated suppression of arthritis and Breg maintenance. To address this, broad-spectrum antibiotic (ABX)-treated mice were given butyrate by oral gavage; this combination of antibiotics is known to ablate the majority of the gut microbiota (Rakoff-Nahoum et al., 2004, Rosser et al., 2014). We found that the suppressive activity of butyrate depended upon the presence of the endogenous gut microbiota, as butyrate supplementation was ineffective at suppressing arthritis in ABX-treated mice (Figure 6A). In support of our previously published results showing that commensal microbiota is important in Breg differentiation, B cells isolated from ABX-treated mice expressed less *Il10* mRNA compared to untreated controls (Rosser et al., 2014), and this defect was not recovered after butyrate supplementation (Figure 6B).

Having established that commensal microbes are required for butyrate to suppress arthritis, we compared the relative abundance of bacteria phyla in the stool of naive, control, and butyrate-supplemented arthritic mice using 16S rDNA amplicon sequencing. Butyrate supplementation induced a shift in the stool microbiota of arthritic mice, favoring a profile that was more similar to naive mice (Figure 6C). A detailed analysis of the bacterial composition revealed an increase in the abundance of the bacterial genera *Allobaculum*, *Bifidobacterium*, and *Rhodosprillaceae*_unclassified in butyrate-supplemented versus control mice (Figure 6D). Members of these bacteria genera have a previously described role in influencing the generation of tryptophan-derived metabolites, a family of ligands implicated in the activation of AhR (Gao et al., 2018). To understand whether changes in bacterial composition following butyrate supplementation altered the level of tryptophan-derived metabolites, we measured these metabolites in the stool of butyrate-supplemented and control mice. There were no differences in the amount of tryptophan, tryptamine, indole, and L-kynurenine in stool samples from butyrate-supplemented compared to control mice (Figures 6E and 6F). Indole-3-acetate and Indole-3-propionate levels were also measured but found to be below the limit of detection in all samples. There was, however, a significant increase in 5-HIAA, the main metabolite of serotonin (Figure 6G), and a significant reduction in the level of the kynurenine-derived metabolite kynurenic acid (KYNA) (Figure 6F).

**Figure 7:**
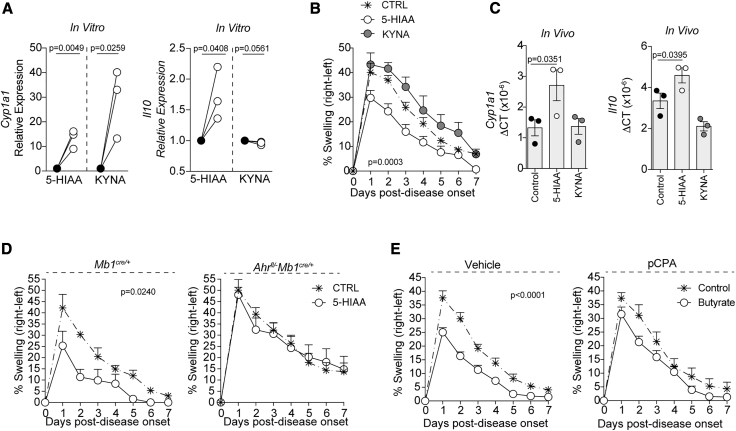
5-Hydroxyindole-3-Acetic Acid Increases *Il10* Transcription by B cells *In Vivo* and *In Vitro* by Acting as a Ligand for AhR (A) Relative expression of *Cyp1a1* and *Il10* in total splenic B cells following 6-h culture with 5-HIAA or kynurenic acid (KYNA) compared to vehicle alone (n = 3, one of two experiments is shown). (B) Mean clinical score of control, 5-HIAA-gavaged, or KYNA-gavaged mice; y axis shows percentage swelling in antigen-injected knee compared to control knee (cumulative n = 8 per group, one representative experiment of two experiments is shown). (C) Bar charts show expression of *Cyp1a1* and *Il10* relative to *β-actin* in splenic B cells isolated from control, 5-HIAA-gavaged, or KYNA-gavaged mice. (D) Mean clinical score of control or 5-HIAA-gavaged *Mb1*^*cre/+*^ mice or *Ahr*^*fl/*^^−^*Mb1*^*cre/+*^ mice; y axis shows percentage swelling in antigen-injected knee compared to control knee (cumulative n = 8 per group, one representative experiment of two experiments is shown). (E) Mean clinical score of control and butyrate-supplemented L-*para*-chlorophenylalanine (PCPA)-treated (tryptophanase inhibitor, TPH) or vehicle-treated mice; y axis shows percentage swelling in antigen-injected knee compared to control knee (cumulative n = 10 per group, one representative experiment of two experiments is shown). Data represent mean ± SE (A, Student’s t test; B, two-way ANOVA; C, one-way ANOVA; D and E, two-way ANOVA).

To directly address how the changes in 5-HIAA and KYNA levels affect AhR-dependent gene transcription in B cells, WT B cells were isolated from naive mice and stimulated with 5-HIAA and KYNA *in vitro*. Unlike KYNA, which only induced *Cyp1a1* induction in B cells, 5-HIAA increased both *Cyp1a1* and *Il10* expression in B cells compared to vehicle-control-treated B cells (Figure 7A). Most importantly, treatment of WT mice with these AhR ligands *in vivo* demonstrated that 5-HIAA, but not KYNA, suppressed arthritis development and increased both *Cyp1a1* and *Il10* transcription in B cells *ex vivo* (Figures 7B and 7C). To examine the role for AhR in the immunosuppressive effect of 5-HIAA, we gavaged *Mb1*^*cre/+*^ mice and *Ahr*^*fl/−*^*Mb1*^*cre/+*^ mice with 5-HIAA. 5-HIAA suppressed arthritis in *Mb1*^*cre/+*^ mice but not in A*hr*^*fl/−*^*Mb1*^*cre/+*^ mice (Figure 7D). Finally, to explore the role of 5-HIAA in the ability of butyrate supplementation to suppress arthritis, mice were treated with the tryptophan hydrolase (TPH) inhibitor L-*para*-chlorophenylalanine (PCPA), which is known to reduce 5-HIAA and serotonin biosynthesis (Welford et al., 2016). In mice treated with PCPA, butyrate supplementation lost its ability to suppress arthritis when compared to vehicle-treated control mice (Figure 7E). Collectively, these data demonstrate that butyrate supplementation increases the production of 5-HIAA, a newly identified AhR ligand in B cells, which mediates the suppressive effect of butyrate supplementation *in vivo*.

## Discussion

Bregs are generated in the periphery in response to bacterially derived and inflammatory signals. Whereas more is understood regarding how inflammation and inflammatory cytokines drive Breg differentiation (Menon et al., 2016, Mizoguchi et al., 1997, Rosser et al., 2014, Yoshizaki et al., 2012), the participation of microbiota in Breg biology remains underexplored. We report that RA patients and arthritic mice have decreased levels of the SCFA butyrate, a microbial metabolite produced by commensal bacteria after dietary fiber fermentation. We also report that butyrate supplementation attenuates disease severity in mice by supporting AhR^+^Breg function and suppressing mature B cell subset differentiation. Based on our results, we propose the hypothesis that butyrate overcomes the need for inflammatory stimuli in Breg differentiation by increasing the availability of the 5-HIAA, which directly activates AhR and *Il10* transcription. This hypothesis needs further exploration and could offer important immunological and physiological groundwork for future therapeutic interventions.

Recent literature has demonstrated that butyrate can alter the function of a wide variety of immune cells (Corrêa-Oliveira et al., 2016). In agreement with this, we found that butyrate supplementation of arthritic mice lowered effector cytokine production by CD11b^+^ splenocytes and enhanced Treg suppressive function upon adoptive transfer. Butyrate has been shown to induce Foxp3^+^ Tregs both directly, by acting as an HDACi (Arpaia et al., 2013), and indirectly, by promoting anti-inflammatory properties in macrophages and dendritic cells by engaging G protein-coupled receptors GPR43 and GPR109A (Gurav et al., 2015, Singh et al., 2014). Building on these findings, we found that Tregs only displayed enhanced suppressive function when isolated from butyrate-supplemented mice with a fully functional Breg compartment. This supports published data demonstrating that Treg homeostasis is altered in mice lacking IL-10-producing B cells (Carter et al., 2011). Here, we also describe a previously unappreciated role for butyrate in altering B cell differentiation and function in both mice and humans with arthritic disease. We found that butyrate levels are reduced in RA patients compared to HCs, and that in RA patients, butyrate levels correlate with IL-10-producing B cell frequency. Interestingly, we observed that in HCs there is a trend for a reduction in butyrate levels in older individuals, which may contribute to changes in autoimmune disease susceptibility in later life. Unlike in humans, there was not a direct correlation between butyrate levels and Breg frequency in mice, likely due to differences in disease chronicity, treatment, and tissue analyzed. However, interrogation of the interaction between butyrate and B cells using a murine model of arthritis demonstrated a sophisticated system whereby butyrate alters AhR-dependent gene transcription, including key B cell differentiation genes and immunoregulatory genes serving to support Breg suppressive function and inhibit B cell maturation. Notably, we found that μMT mice, which lack both regulatory and inflammatory (e.g., GC B cells and plasmablasts) B cells, have equivalent disease severity to WT mice in this model of arthritis, but that chimeric mice, which exclusively lack IL-10-producing B cells, develop exacerbated disease compared to chimeric mice with WT B cells (Carter et al., 2011). This demonstrates a fundamental role for IL-10-producing B cells in suppressing arthritic severity following butyrate supplementation in this model. These data demonstrate for the first time that a microbially derived metabolite can control the balance between regulatory and mature B cell subsets.

Our data determined that butyrate supplementation requires a fully competent endogenous microbiota to exert its anti-arthritogenic capabilities on the B cell compartment. Butyrate supplementation shifted the microbiota to increase relative abundance of *Allobaculum*, *Bifidobacterium*, and *Rhodosprillaceae_unclassified*, genera which have been shown to influence tryptophan metabolism (Desbonnet et al., 2008, Gao et al., 2018, Lamas et al., 2016, Mujahid et al., 2010). One possible explanation for this shift is that butyrate possesses antimicrobial activity that targets pathobionts, creating a niche for the growth of tryptophan-metabolizing species. In the agricultural industry, butyrate is an established component of chicken feeds used to control the growth of pathogenic bacteria (Vahjen et al., 1998). In addition to a direct bactericidal effect, butyrate enhances the microbicidal function of macrophages by altering their metabolism and eliciting the production of anti-microbial peptides, which may control out-growth of pathogenic components of the gut microbiota (Schulthess et al., 2019). Another complementary explanation justifying the observed shift in bacterial communities is that butyrate acts as a nutrient for beneficial bacteria. At present, we cannot exclude that changes observed in the gut microbiota following butyrate supplementation could be the result of reduced inflammation. Unfortunately, due to the intertwined response between microbiota and inflammation, it is difficult to extricate whether the butyrate effect on bacteria is direct, or mediated by cells or other anti-inflammatory mediators. Future studies will be performed to investigate if the effect reported here is due to changes in inflammation or to direct effect on the microbiota. Our findings support the notion that prebiotics supplementation could be used to restrain inflammation in systemic autoimmune disease with no obvious gut-related pathogenesis.

AhR is an environmental sensor detecting both xenobiotic ligands and physiological compounds generated by host cells, microbiota, and diet (Zhou, 2016). Among the microbiota-derived ligands for AhR, an important family are tryptophan-derived metabolites. For example, it has been previously demonstrated that tryptophan is endogenously metabolized into tryptamine and indole-3-acetic acid, which directly bind to AhR (Heath-Pagliuso et al., 1998, Vikström Bergander et al., 2012). More recently, expression of tryptophanase by certain microbiota species has been shown to process tryptophan into indoles and its 3-substituted derivatives, which also act as agonists for AhR (Rasmussen et al., 2016). In addition, L-kynurenine and kynurenic acid, which are produced following metabolism of tryptophan by indoleamine 2,3-dioxygenase (IDO), can also activate AhR in immune cells (Quintana et al., 2008, Seok et al., 2018). In this study, we did not detect any variation in the levels of tryptophan, tryptamine, L-kynurenine, or indole, yet we observed a reduction in kynurenic acid (KYNA). We also found the levels of indole-3-substituted derivatives to be below the limit of detection, suggesting that these pathways are unaffected by butyrate supplementation. Rather, our data suggest an additional mechanism by which 5-HIAA, the main metabolite of serotonin (5-HT), activates AhR in B cells following butyrate supplementation. Similarly to T cells, where it has been shown that different AhR ligands drive either Treg or Th17 differentiation (Quintana et al., 2008), we show that both KYNA and 5-HIAA can activate AhR-dependent gene transcription in B cells, but only 5-HIAA concomitantly induces AhR signaling and *Ill0* transcription in B cells. The production of the tryptophan-derived neurotransmitter 5-HT in the gut is intimately connected with the presence and species of the gut microbiota (Yano et al., 2015). As well as regulating diverse physiological processes in both the brain and gut, 5-HT also has a proposed immune-modulatory function, including the promotion of B cell proliferation, induction of cytokine release by monocytes, and changing in capabilities of dendritic cells to present antigen and activate T cells (Idzko et al., 2004, O’Connell et al., 2006). Here, we determine that 5-HT’s main metabolite 5-HIAA upregulates AhR-dependent gene transcription and *ll10* transcription in B cells and is immunoregulatory in arthritis. Our data support a recently established link between the serotonergic and AhR pathways, showing the efficacy of 5-HT in inducing *Cyp1a1* expression via AhR in intestinal epithelial cells (Manzella et al., 2018). It also adds to accumulating evidence that butyrate can induce 5-HT release by neural enterochromaffin cells in the gut (Reigstad et al., 2015). We suggest that, as well as regulating gut homeostasis and peristalsis, the butyrate-serotonin-AhR axis also acts to influence Breg homeostasis.

The data in this study suggest that gut-microbiota-derived metabolites control many aspects of B cell development and Breg function. Moreover, it suggests that the threshold for Breg induction in response to inflammatory stimuli could potentially be lowered in AhR-activating ligand-rich environments. Future work will be used to establish whether the dynamics of Breg activation is orchestrated by the complementary effects of inflammation and certain microbial-derived stimuli. To date, due to the heterogenous nature of the Breg response, researchers have been unable to ascertain how to harness the suppressive function of these cells for therapeutic intervention. This report addresses this gap and reveals that manipulation of microbial end-products that can be supplemented during dietary interventions could serve this purpose well.

### Limitations of Study

Here, we demonstrate that reduced butyrate levels in RA patients correlate with reduced Breg frequency in peripheral blood. However, as for many human studies, our results are only correlative. One major caveat of our human study was the inability to include patients with active disease (as they have a very low levels of Bregs) as well as patients with other autoimmune diseases. In future studies, we will aim to include RA patients with both active and inactive disease as well as patients with other inflammatory diseases to ascertain whether changes in SCFA are unique to RA or a common feature of all autoimmune diseases. It would also be important to validate our results in another RA cohort of patients, as microbiota composition changes according to geographical region and diet. Moreover, it has been previously shown that the microbiota is not standardized in every animal house, which based on our results, is likely to have significant impacts on the efficacy of butyrate supplementation to suppress severity of arthritis and support Breg function. Future work is therefore necessary to identify particular 5-HIAA-inducing bacterial species that mediate the effect of butyrate supplementation to circumvent these limitations.

## STAR★Methods

### Key Resources Table

**Table:** 

REAGENT or RESOURCE	SOURCE	IDENTIFIER
**Antibodies**		

InVivoMAb CD25, Clone PC-61.5.3	BioXcell	Cat# BE0012, RRID:AB_1107619
AffiniPure Fab Fragment Goat Anti-Mouse IgM, μ chain specific	Jackson ImmunoResearch	Cat# 115-007-020; RRID:AB_2338477
B220 PE, Clone RA3-6B2	BD Bioscience	Cat# 553090; RRID:AB_244282
CD1d BV510, Clone 1B1	Biolegend	Cat# 563189, RRID:AB_2738056
CD3 BV605, Clone 17A2	Biolegend	Cat# 100237; RRID:AB_2562039
CD4 BV605, Clone RM4-5	Biolegend	Cat# 100548; RRID:AB_2563054
CD4 BV711, Clone RM4-5	Biolegend	Cat# 100550; RRID:AB_2562099
CD5 AF647, Clone 53-7.3	Biolegend	Cat# 100614; RRID:AB_2075301
CD8a BV605, Clone 53-6.7	Biolegend	Cat# 100744; RRID:AB_2562609
CD11b BV605, Clone M1/70	Biolegend	Cat# 101257; RRID:AB_2565431
CD11c BV605, Clone N418	Biolegend	Cat# 117334; RRID:AB_2562415
CD19 BV785, Clone 6D5	Biolegend	Cat# 115543; RRID:AB_11218994
CD19 BV785, Clone HIB19	Biolegend	Cat# 302240; RRID:AB_11218596
CD21 APC, Clone 7G6	Biolegend	Cat# 123412; RRID:AB_2085160
CD21 FITC, Clone 7G6	BD Biosciences	Cat# 553818, RRID:AB_395070
CD23 FITC, Clone B3B4	Biolegend	Cat# 101606; RRID:AB_312831
CD24 PE-Cy7, Clone M1/69	Biolegend	Cat# 101822; RRID:AB_756048
CD24 BV421, Clone M1/69	Biolegend	Cat# 101826; RRID:AB_2563508
CD24 PE/Cy7, Clone ML5	BD Biosciences	Cat# 311119, RRID:AB_2072734
CD25 BV510, Clone PC61	Biolegend	Cat# 102041, RRID:AB_2562269
CD38 BV605, HIT2	Biolegend	Cat# 303531, RRID:AB_2561527
CD43 PE/Cy7, Clone S7	BD Biosciences	Cat# 562866; RRID:AB_2737852
CD95 PE/Cy7, Clone Jo2	BD Biosciences	Cat# 557653, RRID:AB_396768
CD138 BV711, Clone 281-2	Biolegend	Cat# 142519; RRID:AB_2562571
CD138 BV605, Clone 281-2	Biolegend	Cat# 142515, RRID:AB_2562336
F4/80 BV605, Clone BM8	Biolegend	Cat# 123133; RRID:AB_2562305
TER-119/Erythroid cells BV605, Clone TER-119	Biolegend	Cat# 116239; RRID:AB_2562447
Ly6C/G BV605, Clone RB6-8C5	Biolegend	Cat# 108440; RRID:AB_2563311
TCRβ BV605, Clone H57-597	Biolegend	Cat# 109241; RRID:AB_2629563
Tim-1 PE, Clone RMT1-4	Biolegend	Cat# 119506; RRID:AB_2232887
CD249 PE, Clone BP-1	BD Biosciences	Cat# 553735; RRID:AB_395018
Blimp-1 AF647, Clone 5E7	Biolegend	Cat# 150004; RRID:AB_2565618
FoxP3 APC, Clone FJK-16	ThermoFisher Scientific	Cat# 17-5773-82; RRID:AB_469457
GL7 PerCP/CY5.5, Clone GL7	Biolegend	Cat# 144610, RRID:AB_2562979
IL-1beta (pro-form) APC, Clone NJTEN3	Thermo Fisher Scientific	Cat# 17-7114-80, RRID:AB_10670739
IL-6 PE, Clone MP5-20F3	Biolegend	Cat# 504504, RRID:AB_31533
IFN-γ APC, Clone XMG1.2	ThermoFisher Scientific	Cat# 17-7311-82; RRID:AB_469504
IL-10 PE, Clone JES5-16E3	Biolegend	Cat# 505008; RRID:AB_315362
IL-10 APC, Clone JES3-19F1	BD Biosciences	Cat# 554707, RRID:AB_398582
IL-17 PE (TC11-18H10.1)	Biolegend	Cat# 506904; RRID:AB_315464
Purified GL7 monoclonal antibody, Clone GL7	ThermoFisher Scientific	Cat# 14-5902-82, RRID:AB_467715
Goat anti-rat IgM secondary Antibody Alexa 647	ThermoFisher Scientific	Cat# A-21248, RRID:AB_2535816
Rabbit anti-Histone H3 (acetyl K27) antibody	Abcam	Cat# ab4729; RRID:AB_2118291
Rabbit anti-Histone H3 antibody	Abcam	Cat# ab1791; RRID:AB_302613
Goat Anti-Rabbit IgG – H&L Polyclonal antibody, HRP conjugated	Abcam	Cat# ab6721; RRID:AB_955447

**Chemicals, Peptides, and Recombinant Proteins**		

Lipopolysaccharide (LPS)	Sigma Aldrich	Cat# L4391
CpG Class B (ODN 2006)	Invitrogen	Cat# L34961
Methylated bovine serum albumin (mBSA)	Sigma Aldrich	Cat# A1009
Immunisation Grade Bovine Type II Collagen	Chondrex	Cat# 20021
Incomplete Freund’s adjuvant (IFA)	Sigma Aldrich	Cat# F5506
Phorbol-12-myristate-13 acetate (PMA)	Sigma Aldrich	Cat# P8139
Ionomycin	Sigma Aldrich	Cat# I0634
DAPI	Sigma Aldrich	Cat# D9542
Brefeldin A	Biolegend	Cat# 420601
2-Mercaptoethanol	ThermoFisher Scientific	Cat# 31350010
RNase-Free DNase set	QIAGEN	Cat# 79254
Sodium Butyrate (for culture)	Sigma Aldrich	Cat# B5887
Sodium Butyrate (for supplementation)	Sigma Aldrich	Cat# 303410
Butyric acid (for HPLC)	Sigma Aldrich	Cat# B103500
L-*para*-chlorophenylalanine (4-Chloro-DL-phenylalanine)	Sigma Aldrich	Cat# C6506
5-Hydroxyindole-3-acetic acid	Sigma Aldrich	Cat# H8878
L-Kynurenine	Sigma Aldrich	Cat# K8625
Kynurenic Acid	Sigma Aldrich	Cat# K3375
Sodium Acetate (for supplementation)	Sigma Aldrich	Cat# S2889
Sodium Propionate (for supplementation)	Sigma Aldrich	Cat# P1880
Propionic acid	Sigma Aldrich	Cat# P1385
Valeric acid	Sigma Aldrich	Cat# 240370
2-Methylbutyric acid	Sigma Aldrich	Cat# 193070
Isobutyric acid	Sigma Aldrich	Cat# I1754
2-Ethylbutyric acid	Sigma Aldrich	Cat# 109959
Isovaleric acid	Sigma Aldrich	Cat# 129542
Lactic Acid	Sigma Aldrich	Cat# 69785
Acetic Acid	Sigma Aldrich	Cat# 45754
3-Methyl-indole	Sigma Aldrich	Cat# M51458
Indole	Sigma Aldrich	Cat# I3408
Indole-3-Carboxaldehyde	Sigma Aldrich	Cat# I29445
Tryptamine	Sigma Aldrich	Cat# 196747
Tryptophan	Sigma Aldrich	Cat# 193747
Indole-3-acetic acid	Sigma Aldrich	Cat# I5148
Indole-3-propionic acid	Sigma Aldrich	Cat# 57400
*N*-(3-Dimethylaminopropyl)-*N*′-ethylcarbodiimide hydrochloride E7750	Sigma Aldrich	Cat# E7750
2-Nitrophenylhydrazine hydrochloride	Apollo Scientific	Cat# OR1939
Diethyl Ether	Fisher Chemical	D/2450/17
Acetonitrile	Sigma Aldrich	271004
Sodium acetate-13C2	Sigma Aldrich	282014
Sodium butyrate-13C4	Sigma Aldrich	488380
Sodium propionate-d5	Cambridge Isotope Labs	DLM-1601

**Critical Commercial Assays**		

Negative CD43- Isolation Kit	Miltenyi Biotec	Cat# 130-049-801
Picopure™ RNA isolation kit	ThermoFisher Scientific	Cat# KIT0204
iScript™ cDNA synthesis kit	Biorad	Cat# 1708891
iQ™ SYBR® green supermix	Biorad	Cat# 1708882
Nextera DNA library preparation kit	Illumina	Cat# FC-121-1030
MinElute PCR purification kit	QIAGEN	Cat# 28004
Pierce™ BCA Protein Assay Kit	ThermoFisher Scientific	Cat# 23225
QIAamp DNA Mini Kit	QIAGEN	Cat# 51306
***BioPulverizer Lysing Matrix E***	MP Biomedical Europe	Cat# 116914050
Taq PCR Core kit	QIAGEN	Cat# 201225
ZymoBIOMICS Microbial Community DNA Standard	Zymo Research	Cat# D6305
Agencourt AMPure XP	Beckman Coulter	Cat# A63881
***Qubit dsDNA HS Assay Kit-500 assays***	ThermoFisher Scientific	Cat# Q32854
***NEBNext Library Quant Kit for Illumina***	New England BioLabs	Cat# E7630L
***High Sensitivity D1000 ScreenTape***	Agilent	Cat# 5067-5584
***Agilent High Sensitivity DNA Reagents***	Agilent	Cat# 5067-4627
***MiSeq Reagent Kit v2 (500-cycles)***	Illumina	Cat# MS-102-2003
***PhiX Control v3***	Illumina	Cat# FC-110-3001

**Deposited Data**		

16 s Data	This paper	PRJNA603680
Raw and analyzed ATAC-seq data – *Ahr*^*fl/-*^*Mb1*^*cre/+*^ and *Mb1*^*cre/+*^ CD19^+^CD21^hi^CD24^hi^ B cells isolated *ex vivo* from control and butyrate treated mice	This paper and Piper et al., 2019	E-MTAB-7525
Raw and analyzed RNA-seq data - *Ahr*^*fl/-*^*Mb1*^*cre/+*^ and *Mb1*^*cre/+*^ CD19^+^CD21^hi^CD24^hi^ B cells isolated *ex vivo* from control and butyrate treated mice	This paper and Piper et al., 2019	E-MTAB-7345

**Experimental Models: Organisms/Strains**		

Mouse, B6(Cg)-*Il10*^*tm1.1Karp*^/J	Prof. Christopher Karp	RRID:IMSR_JAX:014530
Mouse, C57BL/6J	Envigo	N/A
Mouse, DBA/1J	Envigo	N/A
Mouse, *B6.C(Cg)-Cd79a*^*tm1(cre)Reth*^*/EhobJ*	Jackson laboratory	RRID:IMSR_JAX:020505
Mouse, *B6.129-Ahr*^*tm1Bra*^*/J*	Jackson laboratory	RRID:IMSR_JAX:002831
Mouse, *Ahr*^*−/−*^*Mb1*^*cre/cre*^	Prof. Brigitta Stockinger	N/A
Mouse, *Ahr*^*fl/fl*^*R26R FP635*^*fl/fl*^	Prof. Brigitta Stockinger	N/A
Mouse, *Mb1*^*cre/+*^	Generated in house from above strains	N/A
Mouse, *Ahr*^*fl/-*^*Mb1*^*cre/+*^	Generated in house from above strains	N/A
**Mouse, B6.129S2-Ighm**^**tm1Cgn**^**/J**	Jackson laboratory	RRID:IMSR_JAX:002288
Mouse, 129(B6)-*Il10*^*tm1Cgn*^/J	Prof. Fiona Powrie	RRID:IMSR_JAX:004368
**Mouse, B6.SJL-Ptprc**^**a**^**Pepc**^**b**^**/BoyJ**	Prof. Derek Gilroy	RRID:IMSR_JAX:002014

**Oligonucleotides**		

**qPCR primers**	ThermoFisher Scientific; This paper	N/A
***Actb***		
Fwd 5′-AGATGACCCAGATCATGTTTGAG-3′		
Rev 5′-AGGTCCAGACGCAGGATG-3′		
***Cyp1a1***	QIAGEN	Cat#QT00105756
***Il10***	ThermoFisher Scientific; Yanaba et al., 2009	N/A
Fwd 5′-GGTTGCCAAGCCTTATCGGA-3′		
Rev 5′-ACCTGCTCCACTGCCTTGCT-3′		

**Software and Algorithms**		

GraphPad Prism 6	Graphpad Software	https://www.graphpad.com
Flowjo v10.5.0	Flowjo, LLC	https://www.flowjo.com
Limma	Ritchie et al., 2015	https://bioconductor.org/packages/release/bioc/html/limma.html
STAR	Dobin et al., 2013	https://github.com/alexdobin/STAR
HTSeq	Anders et al., 2015	https://htseq.readthedocs.io/en/release_0.11.1/install.html#install
Kallisto	Bray et al., 2016	https://pachterlab.github.io/kallisto/download
EdgeR	Robinson et al., 2010	https://bioconductor.org/packages/release/bioc/html/edgeR.html
Signaling pathway impact analysis	Tarca et al., 2009	https://bioconductor.org/packages/release/bioc/html/SPIA.html
Venny 2.1	*Oliveros, J.C. (2007-2015) Venny. An interactive tool for comparing lists with Venn’s diagrams.*https://bioinfogp.cnb.csic.es/tools/venny/index.html	https://bioinfogp.cnb.csic.es/tools/venny/
Illumina Casava 1.7	Illumina	https://www.illumina.com
Picard Tools	N/A	https://broadinstitute.github.io/picard/
MACS2 v2.1.1.20160309	Zhang et al., 2008	https://github.com/taoliu/MACS
Integrative Genomics Viewer (IGV)	**James T. Robinson, Helga Thorvaldsdóttir, Wendy Winckler, Mitchell Guttman, Eric S. Lander, Gad Getz, Jill P. Mesirov. Integrative Genomics Viewer.** Nature Biotechnology 29, 24–26 (2011).	https://software.broadinstitute.org/software/igv/
Multiple Experiment Viewer (MeV_4_8)	Saeed et al., 2003	http://mev.tm4.org/#/welcome
Mothur V1.35.13	Schloss, P.D., et al., Introducing mothur: Open-source, platform-independent, community-supported software for describing and comparing microbial communities. Appl Environ Microbiol, 2009. 75(23):7537-41	https://www.mothur.org/
R-studio version 3.6.0		https://rstudio.com/
Phyloseq		https://joey711.github.io/phyloseq/

**Other**		

RPMI-1640 media	Sigma Aldrich	Cat# R8758
Red blood cell lysis buffer	Sigma Aldrich	Cat# R7757
Foetal calf serum (FCS)	Biosera	Cat# FB1001/500
Noraml Goat Serum	Vector	Cat# S1000
LIVE/DEAD™ Fixable Blue	Invivogen	Cat# L34961
Vectashield Mounting Medium with DAPI	Vector	Cat# H-1200
Formalin solution, nerutral buffered, 10%	Sigma Aldrich	Cat# HT501320
Penicillin/Streptomycin	Sigma Aldrich	Cat# P0781
eBioscience™ Intracellular fixation & permeabilization buffer set	ThermoFisher Scientific	Cat# P078188-8824-00
Brilliant stain buffer	BD Biosciences	Cat# 563794
eBioscience™ FoxP3 / Transcription Factor Staining Buffer Set	ThermoFisher Scientific	Cat# 00-5523-00
*M. tuberculosis* H37 Ra, desiccated	BD	Cat# 231141
*Cell Lysis Buffer (10x)*	Cell Signaling Technology	Cat# 9803
*Pierce*^*TM*^*ECL Western Blotting Substrate*	ThermoFisher Scientific	Cat# 32106
*SPME Fiber Assembly 75mm CAR/PDMS, Fused Silica 23Ga*	Sigma Aldrich	Cat# 57344-U

### Lead Contact and Materials Availability

Further information and requests for resources and reagents should be directed to and will be fulfilled by the lead contact, Professor Claudia Mauri (c.mauri@ucl.ac.uk). This study did not generate new unique reagents.

### Experimental Model and Subject Details

#### Human Samples

Peripheral blood (PB) and stool samples from healthy adult controls and rheumatoid arthritis patients were obtained with fully informed consent as approved by the London-Bloomsbury and Riverside Research Ethics Committees (IRAS: 191626 and 46584) in accordance with the Declaration of Helsinki. Clinical and demographical data are shown in Table S1.

#### Mice

B6(Cg)-*Il10*^*tm1.1Karp*^/J (IL-10eGFP) mice were as described and given courtesy of Professor Chris Karp (Madan et al., 2009); B6.129S2-Ighm^tm1Cgn^/J (μMT) mice were purchased from Jackson, USA; 129(B6)-*Il10*^*tm1Cgn*^/J (*Il10*^*−/−*^) mice were kindly given courtesy of Professor Fiona Powrie (Kennedy Institute of Rheumatology Oxford University); B6.SJL-Ptprc^a^ Pepc^b^/BoyJ (CD45.1) mice were kindly given courtesy of Professor Derek Gilroy (University College London); *B6.129-Ahr*^*tm1Bra*^*/J* (*Ahr*^*−/−*^)*, Mb1*^*cre/+*^ (given courtesy of Prof. M. Reth) and *Ahr*^*fl/-*^*Mb1*^*cre/+*^ mice were kindly provided by Professor Brigitta Stockinger (Francis Crick Institute): (all mice were on a C57BL/6 background). For detailed information please see Key Resources Table. C57BL/6 WT mice and DBA/1J WT mice were purchased from Envigo, UK. Sex-matched male and female mice between 6-12 weeks of age were used for antigen-induced arthritis experiments. Analysis of sex-dependent effects was not carried out as there is no reported sex-bias in this model. Male DBA/1J mice 8 weeks of age were used for collagen induced arthritis experiments. Mice were housed at 20-24°C, 45%–64% humidity, and at a 12- light/dark cycle. Experimental mice were feed Harlan Teklad pellets 2018 (18% protein) and breeding mice were feed Harlan Teklad pellets 2010 (19% protein). Mice were specific-pathogen free (Health screening (Full-FELASA profile) was performed annually) and bred and maintained at the animal facility at University College London. All experiments were approved by the Animal Welfare and Ethical Review Body of University College London and authorized by the United Kingdom Home Office.

#### Induction of Antigen-Induced Arthritis (AIA)

AIA was induced and assessed as previously described (Carter et al., 2011, Oleinika et al., 2018, Rosser et al., 2014). Briefly, mice were injected subcutaneously (s.c.) at the tail-base with 200 μg of methylated BSA (mBSA; Sigma-Aldrich) emulsified in 100 μL of complete Freund’s adjuvant (CFA); incomplete Freund’s adjuvant (IFA) (Sigma-Aldrich) containing 3mg/mL of Mycobacterium Tuberculosis (Difco). After seven days, mice received an intra-articular (i.a.) injection of 10 μL of phosphate-buffered saline (PBS) containing 200 μg mBSA in the right knee and 10 μL PBS alone in the left knee as a control. Joint size was measured using calipers (POCO 2T; Kroeplin Längenmesstechnik), and swelling was calculated as a percentage increase in size between inflamed and control knee. Affected joints were removed post-mortem, fixed in 10% (w/v) buffered formalin, and decalcified in 5% EDTA. The joints were subsequently embedded in paraffin, sectioned, and stained with hematoxylin and eosin Y (H&E). Briefly, the sections were rehydrated in PBS, stained with hematoxylin, washed, counterstained with eosin Y, then washed and dehydrated in sequentially higher concentrations of ethanol from 75% to 100%. The sections were scanned using the NDP NanoZoomer (Hamamatsu) at 20x magnification and analyzed with the NDP view software. The joint sections were scored blinded: 1, Normal = no damage; 2, mild = minimal synovitis, cartilage loss, and bone erosion limited to discrete foci; 3, moderate = synovitis and erosion present, but normal joint architecture intact; and 4, severe = extensive erosion and joint architecture disrupted.

#### Induction of Collagen Induced Arthritis (CIA)

CIA was induced and assessed as previously described (Brand et al., 2007). Briefly, 8-week-old male DBA/1J mice were injected intra-dermally (i.d.) at the tail-base with 100 μg of Bovine Collagen Type II (Chondrex) emulsified in 50 μL complete Freund’s adjuvant (CFA); incomplete Freund’s adjuvant (IFA) (Sigma-Aldrich) containing 4mg/mL of Mycobacterium Tuberculosis (Difco). The clinical severity of arthritis was graded as follows: 0, normal; 1, slight swelling and/or erythema; 2, pronounced edematous swelling; 3, pronounced edematous swelling plus joint rigidity; and 4, laxity. Each limb was graded, allowing a maximal clinical score of 16 for each animal. All clinical evaluations were performed in a blinded manner.

### Method Details

#### Short-chain Fatty Acid Supplementation

1 week prior to the induction of arthritis the drinking water of mice was supplemented with sodium acetate, sodium propionate or sodium butyrate (all 150mM; Sigma-Aldrich) and changed every 3 days as described (Smith et al., 2013). A control group received sodium chloride. Mice were maintained on short-chain fatty acids (SCFAs) throughout the duration of the experiment. For RNA-seq and ATAC-seq analysis, mice were gavaged daily with 500mg/kg of sodium butyrate to reduce variation caused by individual differences in daily water intake. Control mice received a gavage of 500mg/kg of sodium chloride. For antibiotic-treated experiments, one week prior to induction of arthritis, vancomycin (500mg/L;Sigma-Aldrich), Neomycin (1g/L; Sigma-Aldrich), and Metronidazole (1g/L;Sigma-Aldrich) were added to drinking water as described (Rosser et al., 2014). Untreated and treated mice were then gavaged daily with 500mg/kg of sodium butyrate or sodium chloride as a control. Mice were maintained on antibiotics throughout the duration of the experiment. For L-*para*-chlorophenylalanine (PCPA) experiments, mice were supplemented with butyrate as described above and gavaged daily with PCPA (4mg per mouse) in a suspension of 0.5% methyl cellulose and 0.01% Tween 80. Control mice received vehicle alone. For Treg depletion experiments, mice were supplemented with butyrate as described above and injected intra-peritoneally with 250 μg of anti-CD25 (PC-61.5.3) or appropriate isotype control on two days prior to and on the day of the commencement of butyrate-supplementation, two days prior and on the day of the subcutaneous injection, and two days prior to and on the day of intra-articular injection.

#### Gavage with 5-Hydroxyindole-3-acetic Acid and Kynurenic Acid

Mice were gavaged daily from 1 week prior to the induction of arthritis and throughout the experiment with either 5-Hydroxyindole-3-acetic acid (5-HIAA, 0.5mg per mouse) or kynurenic acid (KYNA, 0.125mg per mouse) dissolved in oil. Control mice received vehicle alone.

#### Generation of Chimeric Mice

Chimeric mice, and appropriate controls, were generated as previously described (Carter et al., 2011, Rosser et al., 2014). Recipient μMT mice received 800cGy gamma-irradiation via a caesium source. 5 h following irradiation, recipients received 10x10^6^ donor bone marrow cells. To generate mice in which the absence of IL-10 was exclusively restricted to B cells, WT mice were reconstituted with mixture of bone marrow consisting of 80% from μMT (B cell deficient) with 20% from *Il10*^*−/−*^ mice. Control mice received 80% from μMT and 20% bone marrow from WT mice (to give a normal B cell compartment). To generate CD45.2^+^AhR^−/−^CD45.1^+^ congenic chimeric mice, WT mice were reconstituted with 10x10^6^ donor bone marrow cells containing 50% from CD45.1^+^ WT mice and 50% from CD45.2^+^ *Ahr*^*−/−*^ mice. Chimeras were left to fully reconstitute over at least 8 weeks before use in AIA experiments.

#### Cell Preparation

Spleens and inguinal lymph-nodes were dissected post-mortem and mashed through a 70 μm cell strainer (BD Biosciences), as previously described (Carter et al., 2011, Oleinika et al., 2018, Rosser et al., 2014). For splenocytes suspensions, erythrocytes from spleens were lysed using Red Cell Lysis Buffer (Sigma-Aldrich). Total B cells were negatively purified by magnetic separation, according to manufacturer’s instructions (Miltenyi Biotec). IL-10eGFP^+^CD21^hi^CD24^hi^ Bregs were isolated from IL-10eGFP reporter mice and CD19^+^CD21^hi^CD24^hi^ B cells were isolated *Mb1*^*cre/+*^, *Ahr*^*fl/-*^*Mb1*^*cre/+*^, WT *or Ahr*^*−/−*^ mice by cell sort. Lymphocytes were analyzed at day 7 post-disease onset unless otherwise stated. Peripheral blood mononuclear cells (PBMC) from RA patients and healthy controls were prepared by density gradient centrifugation using Ficoll Plaque™ plus (GE Healthcare).

#### *In Vitro* Cell Culture

For assessment of murine IL-10 production, B cells from either male or female mice were negatively purified by magnetic separation (Miltenyi Biotic) (purity < 95%–98%) and cultured for 48 h with LPS (1 μg/mL; Sigma-Adrich), with anti-IgM added for the last 24 h of culture (10 μg/mL; Jackson ImmunoResearch) and supernatants from B cell cultures were harvested and analyzed for cytokines using standard sandwich ELISA kit (IL-10; R&D systems) and performed according to manufacturer’s instructions. For assessment of *Cyp1a1* and *Il10* induction by PCR, B cells were negatively purified by magnetic separation and cultured for 6 h with 5-HIAA (10 μM; Sigma-Adrich) or sodium butyrate (500 μM; Sigma-Adrich). For assessment of inflammatory cytokine production, total lymphocytes were stimulated overnight with 50ng/mL PMA and 250ng/mL Ionomycin. Supernatants were harvested and analyzed for cytokine production using the LEGENDplex Mouse Inflammation Panel (Biolegend) and performed according to manufacturer’s instructions.

#### qPCR

RNA from isolated B cells was extracted using Arcturus Picopure RNA isolation kit (ThermoFisher Scientific) and RNA was reverse transcribed using an iScript cDNA synthesis kit (Bio-Rad), according to manufacturer’s instructions. qPCR was carried out on the cDNA samples using iQ SYBR® Green Supermix (Bio-Rad), according to manufacturer’s instructions. Primers were used at a final concentration of 0.5 μM. A Quantitect primer for *Cyp1a1* was purchased from QIAGEN. Primers for *β-Actin* were custom designed with the following sequences: *Act* Forward (5′-AGATGACCCAGATCATGTTTGAG-3′); *Act* Reverse (5′- AGGTCCAGACGCAGGATG-3′) and *Il10* as previously described (Yanaba et al., 2009). qPCR data were calculated as the ratio of gene to *β-*Actin expression by the relative quantification method.

#### Flow Cytometry and Cell Sorting

Flow cytometry was performed as previously described using directly conjugated monoclonal antibodies (abs) and dead cells were excluded using a live/dead discrimination dye (Thermo Scientific) prior to analysis, as previously described. Briefly, for extracellular multi-color analysis, cells were stained at 4°C for 20min as described (Carter et al., 2011, Oleinika et al., 2018, Rosser et al., 2014). For *ex vivo* analysis of intra-nuclear transcription factors, following extracellular staining, cells were fixed for 20min with Fix Perm (eBioscience) and following permeabilization were incubated with intracellular Abs. For detection of IL-17 and IFN-γ cells were suspended at 5x10^6^ cells/mL in complete medium with PMA (50ng/mL; Sigma-Aldrich), Ionomycin (250ng/mL; Sigma-Aldrich) and GolgiPlug (BD Biosciences) for 4 h. For detection of IL-10 by murine B cells, splenocytes were suspended at 5x10^6^ cells/mL in complete medium with PMA (50ng/mL; Sigma-Aldrich), Ionomycin (250ng/mL; Sigma-Aldrich) and Brefeldin A (BD Biosciences) for 4 h. For detection of IL-10 by human B cells, total PBMC were cultured with CpGB ODN2006 (1 μM; Invivogen) for 72 h prior to stimulation with PMA (50ng/mL), Ionomycin (250ng/mL) and Golgiplug for 4 h. Cells were then stained with surface markers followed by permeabilization and incubation with intracellular Abs. IL-10eGFP expression, was analyzed *ex vivo* without fixation, and 4,6-diamidino-2-phenylindole (DAPI) (1 μg/mL; Sigma-Aldrich) was added just before acquisition. Splenocytes from WT mice were used as a control to set gates for IL-10eGFP^+^ cells. Flow cytometric data were collected on an LSRII or LSR Fortessa (BD PharMingen) using FACS Diva software. Data were analyzed using Flowjo (Tree Star). For adoptive transfer experiments, B cell subsets were stained with antibodies against CD19, CD21, and CD24, dead cells were excluded by the use of DAPI (1 μg/mL) and cells were sorted on a FACS aria (BD PharMingen). For RNA-seq and ATAC-seq experiments, a dump channel using antibodies against CD3, CD4, CD8a, CD11b, CD11c, F4/80, Ly6C/G, erythroid cells (TER-119) and TCRγδ (H57-597) was included. Sort purity of B cell subpopulations was routinely > 95%.

#### Adoptive Transfer of Bregs

B cells were isolated from the spleen of either butyrate-supplemented mice or control IL-10eGFP reporter mice seven days post-disease onset and stained with antibodies against CD19, CD21, and CD24. B cell subsets were FACS sorted using gates drawn according to previous reports (Carter et al., 2011, Oleinika et al., 2018, Rosser et al., 2014). IL-10eGFP^+^CD19^+^CD21^hi^CD24^hi^Bregs (2.3 × 10^5^) were transferred intravenously into recipient mice on the day of intra-articular injection. The control group (no transfer) received a PBS injection. For congenic transfer experiments, CD45.2^+^CD19^+^CD21^hi^CD24^hi^ B cells (0.75 × 10^6^) were transferred from control mice or mice that have received butyrate-supplementation into recipient CD45.1^+^ mice.

#### Adoptive transfer of Tregs

CD3^+^CD4^+^CD25^+^ were isolated from butyrate-supplemented and control *Mb1*^*cre/+*^ and *Ahr*^*fl/-*^*Mb1*^*cre/+*^ mice seven days post-disease onset and CD3^+^CD4^+^CD25^+^Tregs (2x10^5^) were transferred intravenously into recipient WT mice on the day of intra-articular injection. The control group (no transfer) received a PBS injection.

#### Immunofluorescence

Spleens were dissected and embedded into optimal cutting temperature compound (OCT, Tissue-Tek) and snap-frozen for cryo-sectioning (6 μm). Slides were incubated in 100% ethanol to fix for 5-10min (4°C), followed by rehydration in PBS for 5min (4°C). The sections were blocked with 10% normal goat serum and 0.3% TX-100 (20 min at RT) and then incubated with primary antibodies for 2 h at RT. Primary antibodies: rat anti-mouse GL7 (Thermo Fisher), B220-PE (BD). Secondary antibody: AF647 – conjugated anti-rat IgM (1 h at RT). The slides were mounted in Vectashield with DAPI (Vector Labs). Whole slide fluorescent images (20x) were taken on a Zeiss Axio Scan Z1 microscope using the 365 nm LED to detect DAPI staining in the nuclei, the 470 nm LED for GFP detection (IL-10), the 555 nm LED for PE detection (B220) and the 625 nm LED for the detection of Alexa fluor 647 (GL7). Scans were analyzed using Leica Software and Fiji (ImageJ).

#### Extraction and Derivation of Short-chain Fatty Acids from Mouse Stool Pellets

Individual stool pellets were weighed into clean Eppendorf tubes and homogenized in 1ml of 50% methanol. After centrifugation at 13,000xg for 5mins to remove particulate matter, 200 μL of the clear supernatants were derivatized as previously reported (Torii et al., 2010). Briefly, the clear supernatants were spiked with 2-ethylbutyric acid as an internal standard and the mixture incubated with 2-Nitrophenylhydrazine hydrochloride (NPH) at 60°C for 20mins, with 1-Ethyl-3-(3-dimethylaminopropyl) carbodiimide (EDC) in pyridine as catalysts. The reaction was then terminated and color allowed to develop by the addition of potassium hydroxide in methanol, followed by incubation at 60°C for a further 20mins. After cooling, the mixture was acidified by the addition of phosphoric acid and the derivatized fatty acids extracted into diethyl ether. After drying down the ether extracts under a gentle stream of nitrogen gas, the resulting fatty acid hydrazides were dissolved in methanol for high performance liquid chromatography (HPLC) analysis.

#### Analysis of Short-chain Fatty Acid Hydrazides by High Performance Liquid Chromatography

Separation of short-chain fatty acid (SCFA) hydrazides was performed by injecting 25 μL onto on a C8 Hypersil MOS2 column (250 × 4.6mm, 5 μm particle size) and eluting using a linear gradient of 20%–90% acetonitrile against water over 17min at a flow rate of 1.5ml/min. Compounds eluting from the column were monitored by UV/Vis absorption using a measurement wavelength of 400nm, and quantitated by integration of peak area. Standard curves (20.0-0.1 μM) were constructed using pure compounds as follows: Succinic acid (R_t_ 3.92 min), Lactic acid (R_t_ 4.72 min), Acetic acid (R_t_ 5.067 min), propionic acid (R_t_ 6.173 min), iso-butyric acid (R_t_ 7.387 min), butyric acid (R_t_ 7.587 min), 2-methylbutyric acid (R_t_ 8.733 min), isovaleric acid (R_t_ 8.947 min), n-valeric acid (R_t_ 9.24 min)_,_ hexonoic acid (R_t_ 10.707 min), 2-ethylbutyric acid (R_t_ 9.88 min) hexanoic acid (R_t_ 10.707 min), Pyruvic acid (R_t_ 12.773).

#### Analysis of Short-chain Fatty Acid by Gas Chromatograph Mass Spectrometry

Plasma samples were analyzed as previously described with modifications to measure concentration of unlabelled acetate propionate and butyrate (Moreau et al., 2004). Internal standard solution (1,2-^13^C-sodium acetate 40 μmoles, d_5_-sodium propionate 4 μmoles, 1,2,3,4-^13^C-sodium butyrate 2 μmoles), was added to 200 μL plasma. Samples were deproteinized with 20ul 20% w/v sulfosalicylic acid and centrifuged for 5mins at 15000 rpm. A 1cm Carboxen/PDMS coated solid-phase microextraction fiber (SPME, Supelco) was inserted into 120 μL supernatant, and analytes adsorbed for 5min. The SPME fiber was then analyzed by gas chromatography mass spectrometry GC/MS, (Thermo Trace GC/DSQ II or Agilent 6890 with 5973N). The inlet temperature was 270°C, and samples were desorbed for 6min and analyzed on HP-FFAP capillary column (25 m x 0.2mm, 0.33 μm film thickness, 0.9ml/min helium carrier flow) at a 1:10 split ratio. The temperature program was: 80°C for 3min, increased to 170°C at 15°C·min−1 and 170°C held for 1min. Ions at m/z 60 were monitored for acetate and butyrate, m/z 62 for 1,2-^13^C- acetate and 1,2,3,4-^13^C-sodium butyrate, m/z 74 for propionate and m/z 79 for d_5_- propionate. Concentrations were calculated by peak area ratios with the respective internal standards.

#### Extraction of Indoles, Kynurenine and Kynurenic Acid from Mouse Faecal Pellets

Individual faecal pellets were weighed into clean Eppendorf tubes and homogenized in 200 μL of methanol. After centrifuging at 13,000xg for 5min to remove particulate matter, the clear supernatants were diluted 1 in 10 in methanol and subject to high performance liquid chromatography (HPLC) analysis.

#### Analysis of Indoles by High Performance Liquid Chromatography

Separation of indoles was performed by injecting 20 μL onto on an ODS Hypersil column (150 × 4.6mm, 3 μm particle size) and eluting using a linear gradient of 5%–100% acetonitrile in 10mM ammonium formate buffer, pH 3.5 over 20min at a flow rate of 0.8ml/min. Compounds eluting from the column were monitored by both fluorescence detection (λ_ex_: 275nm, λ_em_: 352nm) as well as by UV/Vis absorption using an online PDA detector (scanning 200-650nm), and quantitated by integration of peak area. Standard curves (20.0-0.1 μM) were constructed using pure compounds as follows: tryptophan (R_t_ 6.97 min), tryptamine (R_t_ 13.04 min), indole (R_t_ 14.41 min), indole-3-acetic acid (R_t_ 11.42 min), indole-3-propionic acid (R_t_ 13.7), 3-methylindole (R_t_ 15.87 min), indole 3-carboxaldehyde (R_t_ 10.96 min), 5-Hydroxyindole-3-acetic acid (R_t_ 8.09 min).

#### Analysis of L-Kynurenine and Kynurenic Acid by High Performance Liquid Chromatography

Separation of L-Kynurenine and Kynurenic Acid was performed by injecting 25 μL onto on an ODS Hypersil column (150 × 4.6mm, 3 μm particle size) and eluting using a linear gradient of 1%–10% acetonitrile in 10mM ammonium formate buffer, pH 3.5 over 17min at a flow rate of 0.8ml/min. Compounds eluting from the column were monitored by both fluorescence detection (λ_em_: 364nm, λ_em_: 480nm for 7.5 min followed by λ_em_: 330nm, λ_em_: 390nm for the remainder of the run) as well as by UV/Vis absorption using an online PDA detector (scanning 200-650nm), and quantitated by integration of peak area. Standard curves were constructed using pure compounds as follows: L-Kynurenine (R_t_ 5.97 min) and Kynurenic Acid (R_t_ 10.6 min).

#### Western Blot

B cells were negatively purified from WT mice and culture for 18 h with 500 μM of butyrate (Sigma-Aldrich). B cells were the lysed and protein from 10x10^6^ cells was resolved by SDS-PAGE, transferred to polyvinylidene fluoride (PVDF) membranes (Amersham), and blotted using anti-H3K27ac (Abcam) and anti-pan-H3 (Abcam). Bound antibodies were revealed with HRP-conjugated species-specific secondary antibodies using ECL substrate (Amersham).

#### 16S rDNA Sequencing

20-50mg of faecal material was extracted using the QIAmp DNA mini kit (QIAGEN). Extraction was carried out as per the manufacturer’s protocol with an additional cell disruption step by bead beating using lysing matrix E (MP Biolmedicals) at 50 Hz for 1min (Tissuelyser LT, QIAGEN). Two negative extraction controls were included. Barcoded primers spanning the V3-V4 region of the 16S rRNA gene were designed according to (Kozich et al., 2013) to include Illumina adaptor, an 8 nucleotide barcode sequence, a 10 nucleotide pad sequence, a 2 nucleotide linker, and a gene-specific primer:: 341F-CCTACGGGNGGCWGCAG or 805R-GACTACHVGGGTATCTAATCC. (Sigma-Aldrich, Dorset, UK). Extracted DNA samples were amplified with different barcode combinations using the Taq Core PCR kit (QIAGEN) as per manufacturer’s instructions with forward and reverse primers at 0.5μM each. A Microbial Community Standard (Zymo Research) of known bacterial composition was also amplified to assess any bias and error rates. The PCR cycling conditions were as follows: initial denaturation at 95°C for 3min, 30 cycles of 95°C for 30sec, 54°C for 30sec, 72°C for 10min and a final extension at 72°C for 10min. PCR products were purified with AMPure beads (0.7x, Beckman Coulter) and quantified using the Qubit dsDNA High Sensitivity Assay Kit (ThermoFisher). Samples were then pooled to create libraries with approximately equal concentrations of 16S rRNA amplicons from each sample. The pooled library was quality and quantity checked using the High Sensitivity D1000 ScreenTape assay (Agilent Technologies) and a NEBNEXT library quantification kit (New England Biolabs). The pooled library was spiked with 10% PhiX (Illumina) and sequenced on an Illumina MiSeq using the Reagent Kit V2 with 500 cycles (Illumina) and custom primers as previously described (Kozich et al., 2013). The open-source software Mothur V1.35.13 was used for initial bioinformatic analysis of the sequencing data (Kozich et al., 2013). Raw sequencing data was demultiplexed and processed according to the online Mothur SOP (Schloss et al., 2009). Sequences were trimmed and those with ambiguous bases were discarded. Suspected chimeric sequences were identified using VSEARCH (Rognes et al., 2016) and removed. Phylogenetic identification of each OTU was achieved by aligning sequences to the SILVA 16S alignment database (v128) (Quast et al., 2013). Sequences that did not meet a 97% similarity threshold were discarded of. Sample reads were rarefied to 50,000 reads prior to further analysis. OTU values generated by Mothur were further analyzed using R-studio (phyloseq) or GraphPad Prism (la Jolla, USA) v. 400 Software for Apple Mac.

#### RNA sequencing

Splenic CD19^+^CD21^hi^CD24^hi^B cells were isolated from butyrate-supplemented and control *Mb1*^*cre/+*^ and *Ahr*^*fl/-*^*Mb1*^*cre/+*^ mice at day 7 post-disease onset. Dead cells were excluded using DAPI. Total RNA was isolated from these populations using the Picopure RNA isolation kit (ThermoFisher Scientific), according to manufacturer’s instructions. 60bp single reads were sequenced on 3 lanes of an Illumina hiseq. 130-500ng of total RNA was fragmented followed by reverse transcription and second strand cDNA synthesis. The double strand cDNA was subjected to end repair, A base addition, adaptor ligation and PCR amplification to create libraries. Libraries were evaluated by Qubit and TapeStation. Sequencing libraries were constructed with barcodes to allow multiplexing of samples in 3 lanes. Around 23-43 million single-end 60-bp reads were sequenced per sample on an Illumina HiSeq 2500 V4 instrument. Poly-A/T stretches and Illumina adapters were trimmed from the reads using cutadapt. Resulting reads < 30bp were discarded. Reads were mapped to the *Mus musculus* GRCm38 reference genome using STAR (Dobin et al., 2013). Gene annotations were applied from Ensembl (EndToEnd option and outFilterMismatchNoverLmax was set to 0.04). Gene expression levels were quantified using htseq-count (“HTSeq,” n.d.) (Anders et al., 2015), using the gtf above. Transcripts per million (TPM) values were estimated independently using Kallisto (Bray et al., 2016).

#### Bioinformatic Analysis of RNA sequencing Data

Differential expression analysis was carried out using the default settings of the edgeR algorithm (Robinson et al., 2010). Genes were considered differentially expressed when the false discovery rate (FDR) adjusted *p-value*s were < 0.05. Signaling Pathway Impact Analysis (SPIA) (Tarca et al., 2009) was used to detect significantly over- or under-expressed pathways, with the Kyoto Encyclopedia of Genes and Genomes (KEGG) Pathways database (Kanehisa et al., 2016) employed as a reference. The full mouse genome was used as background for enrichment.

#### ATAC-seq

Splenic CD19^+^CD21^hi^CD24^hi^B cells were isolated from butyrate-supplemented and control *Mb1*^*cre/+*^ and *Ahr*^*fl/-*^*Mb1*^*cre/+*^ mice as above for RNA-seq. After sorting, 40,000 cells were washed with 1xPBS (10% FCS). The cell pellet was prepped for sequencing by using the Nextera DNA library preparation kit (Illumina). Briefly, 10.5 μL nuclease free water, 12.5 μL 2x Transposase buffer, 2 μL transposase and 0.25 μL digitonin (0.05%) per reaction were added to the cell pellets. Cells were incubated at 37°C for 30mins. DNA was then purified using a MinElute PCR purification kit (QIAGEN), according to manufacturer’s instructions. Following DNA purification, 1μl of eluted DNA was used in a qPCR reaction to estimate the optimum number of amplification cycles. Library amplification was followed by solid phase reversible immobilization (SPRI) size selection to exclude fragments larger than 1,200bp. DNA concentration was measured with a Qubit fluorometer (Life Technologies). Library amplification was performed using custom Nextera primers. The libraries were sequenced by the Biomedical Sequencing Facility at CeMM using the Illumina HiSeq4000 platform and the 50bp single-end configuration.

#### Bioinformatic Analysis of Chromatin Accessibility Data

Sequenced reads were trimmed for adaptor and Nextera sequences with *skewer* and reads were mapped to mm10 reference genome using bowtie2 v2.2.4 with the “-very-sensitive” parameter. Duplicate reads were marked with *sambamba* and reads were filtered for quality above 30. Peaks for ATAC-seq samples were called with *MACS2* version 2.1.1.20160309 using the “-nomodel -extsize 147” parameters. A consensus region set was generated by creating a union of all samples’ summits extended by 250bp, from where blacklisted regions defined by the *ENCODE* project for mm10 were filtered out. Quantification of chromatin accessibility for each sample was performed by counting the number of reads overlapping each regulatory element for each sample. *DESeq2* was used to compare either the effect of genotype or butyrate treatment on the chromatin landscape, and for visualization we created a normalized chromatin accessibility matrix by normalizing for the regulatory elements’ length and GC content using the R package *cqn*. To assess transcription factor activity, we employed *ChromVar* by fixing a 500bp window on the center of the regulatory elements and using the JASPAR2016 database, computing deviations and their scores, followed by differential variability for genotype and treatment with default parameters.

### Quantification and Statistical Analysis

#### Statistical Analysis

Heatmap analyses for microarray, RNA-seq and ATAC-seq datasets were performed using Multiple Experiment Viewer (MeV_4_8) software (Saeed et al., 2003). Hierarchical clustering was applied to genes using average linking clustering with the Euclidean distance metric. All data is expressed as Mean ± SE, representative of one experiment. Total *n* number is indicated in the figure legend. For *in vivo* studies power calculations were performed on data showing mean maximum wild type arthritic knee swelling of 2mm with a standard deviation of 0.39 mm, and an expected test group arthritic knee swelling of 1.4mm. Group sizes of 3 mice or above were sufficient to reach a statistical power of at least 80%. Mice were assigned at random to treatment groups for all mouse studies, and where possible mixed among cages. Clinical and histological scoring was performed in a blinded fashion. Mice that developed adverse reactions to protocols were excluded from datasets. Statistical significance was determined: using unpaired t tests (comparison of two groups), using Mann-Whitney tests (comparison of two groups, non-parametric data), Spearman’s correlation, one-way ANOVA (comparison of three or more groups) or two-way ANOVA (murine clinical data only). All data met the assumption of statistical tests and variance was similar between groups that were statistically compared and were corrected for multiple comparisons or differences in variance. Results were considered significant at p ≤ 0.05. Statistical tests were carried out using GraphPad Prism (la Jolla, USA) v. 400 Software for Apple Mac.

### Data and Code Availability

The RNA-seq and ATAC-seq datasets generated during this study are available at ArrayExpress (accession numbers E-MTAB-7345; E-MTAB-7525). The 16S datasets generated during this study are available at NCBI Sequence Read Archive (accession number PRJNA603680).

## Supplemental Information

Document S1. Figures S1–S7 and Tables S1–S3

Document S2. Article plus Supplemental Information
