# Supplementary material for: Microbiota-Derived Metabolites Suppress Arthritis by Amplifying Aryl-Hydrocarbon Receptor Activation in Regulatory B Cells
Source: Cell Metab. 2020 Apr 7;31(4):837–851.e10. doi: 10.1016/j.cmet.2020.03.003 (PMC7156916; doi:10.1016/j.cmet.2020.03.003)
Supplement: Document S1. Figures S1–S7 and Tables S1–S3 [file mmc1.pdf]

Cell Metabolism, Volume *31*

Supplemental Information

Microbiota-Derived Metabolites Suppress Arthritis by Amplifying Aryl-Hydrocarbon Receptor Activation in Regulatory B Cells

Elizabeth C. Rosser, Christopher J.M. Piper, Diana E. Matei, Paul A. Blair, André F. Rendeiro, Michael Orford, Dagmar G. Alber, Thomas Krausgruber, Diego Catalan, Nigel Klein, Jessica J. Manson, Ignat Drozdov, Christoph Bock, Lucy R. Wedderburn, Simon Eaton, and Claudia Mauri

Cell Metabolism, Volume *31*

Supplemental Information

Microbiota-Derived Metabolites Suppress Arthritis by Amplifying Aryl-Hydrocarbon Receptor Activation in Regulatory B Cells

Elizabeth C. Rosser, Christopher J.M. Piper, Diana E. Matei, Paul A. Blair, André F. Rendeiro, Michael Orford, Dagmar G. Alber, Thomas Krausgruber, Diego Catalan, Nigel Klein, Jessica J. Manson, Ignat Drozdov, Christoph Bock, Lucy R. Wedderburn, Simon Eaton, and Claudia Mauri

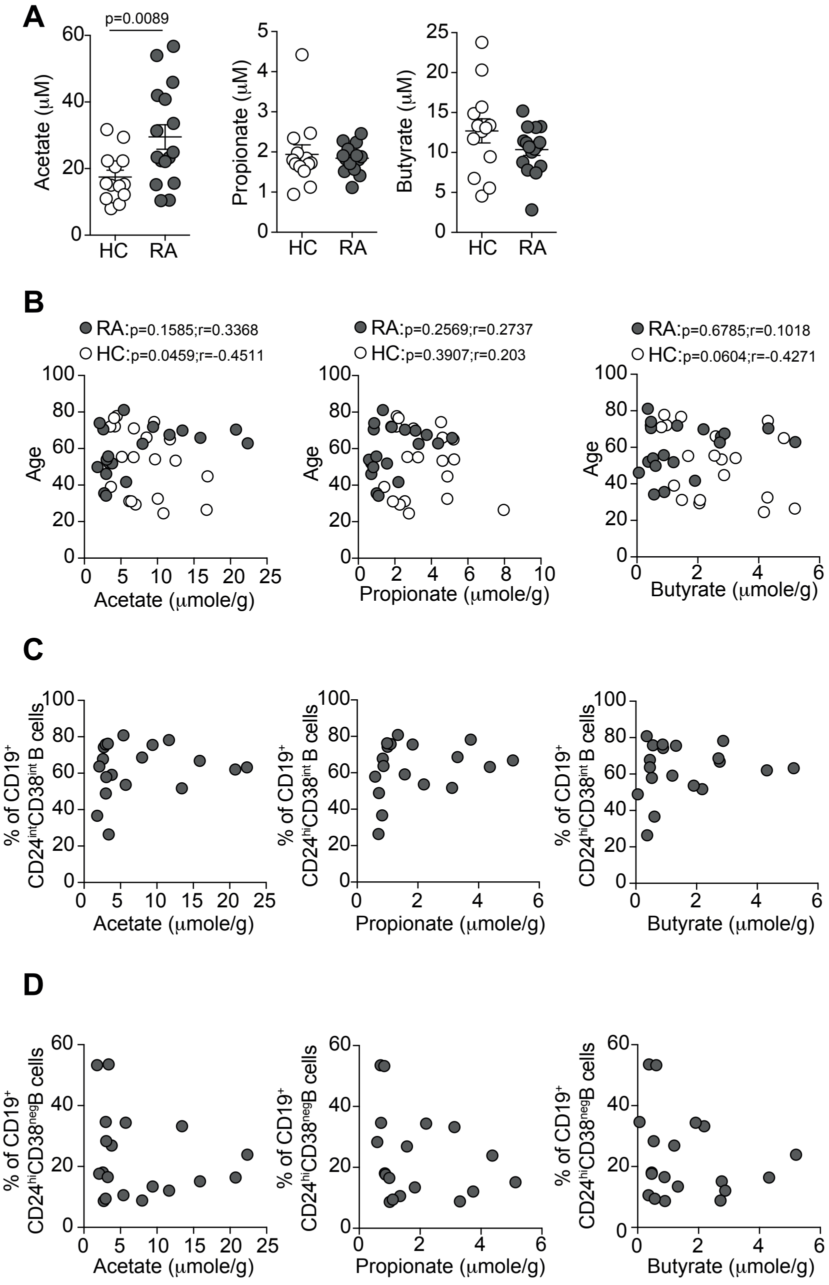

Supplementary Figure 1. There is no association between SCFA levels and mature B cells or memory B cells (Related to Figure 1). (A) Representative histograms show level of acetate, propionate and butyrate in sera of healthy individ-uals (n=13) and individuals with rheumatoid arthritis (RA) (n=14). (B) Scatter plots show correlation between the levels of acetate, propionate and butyrate in faeces and age at time of sample (HC, n=20; RA, n=19). (C) Scatter plots show correlation between the levels of acetate, propionate and butyrate in the faeces and the frequency of CD19$^+$CD24^{int}CD38^{int} (mature naive B cells) in the peripheral blood of patients with RA (n=19). (D) Scatter plots show correlation between the levels of acetate, propionate and butyrate in the faeces and the frequency of CD19$^+$CD24^{hi}CD38^{neg} (memory B cells) in the peripheral blood of patients with RA (n=19). A, Data represent mean ± SE; (A, Student's t test; B-D, Pearson's correlation).

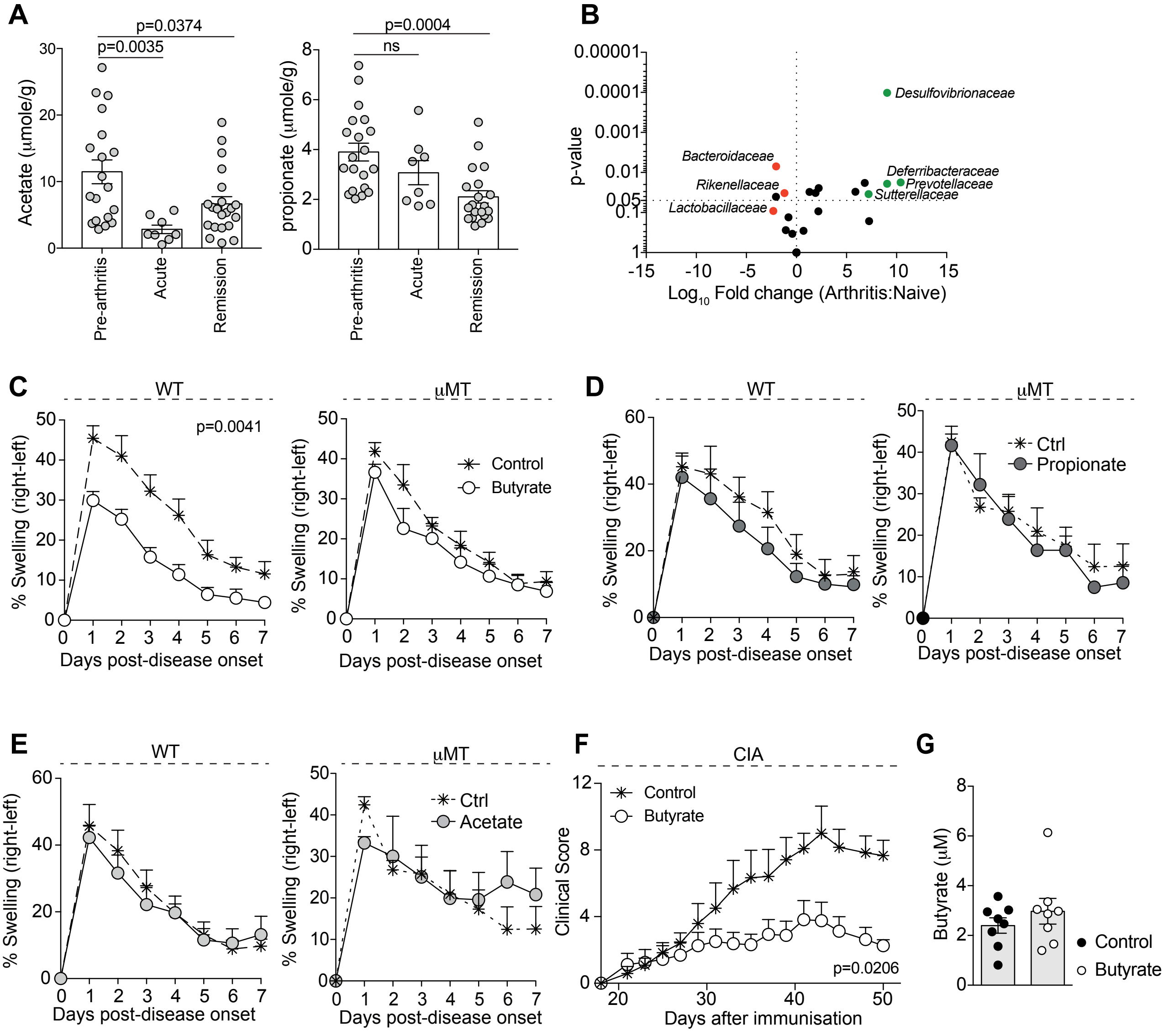

Supplementary Figure 2. Supplementation with Acetate or Propionate does not suppress the development of antigen induced arthritis (Relative to Figure 2). (A) Concentration of acetate and propionate in faeces from WT mice pre-arthritis (n=23), with acute arthritis (n=8) and in remission from arthritis (n=18) as measured by high-performance liquid chromatography (cumulative data are shown). (B) Volcano plot shows fold change between bacterial families in the faeces of naïve mice compared to arthritic mice at day 3 post-disease onset (n=4 per group). (C) Mean clinical score of control (cumulative n=25) and butyrate-supplemented WT mice (cumulative n=24) (one representative experiment of six experiments is shown) or μMT mice (control, cumulative n=7; butyrate, cumulative n=9). (D) Mean clinical score of control and propionate-supplemented WT mice (left) or μMT mice (right) (WT: n =8, μMT: n=6 per group); y axis shows percent-age swelling in antigen-injected knee compared to control knee (one of two experiments is shown). (E) Mean clinical score of control and acetate-supplemented WT mice (left) or μMT mice (right) (cumulative n=10 per group); y axis shows percentage swelling in antigen-injected knee compared to control knee (one representative experiment of two experiments is shown. (F) Mean clinical score following induction of collagen-induced arthritis in control of butyrate-supplemented WT mice (cumulative n=8 per group). (G) Butyrate levels in the sera butyrate-supplemented and control mice (n=8 per group, cumulative data are shown). Data represent mean ± SE. (A, Student's t test; B, multiple unpaired t tests; C-F, two-way ANOVA; G, Student's t test).

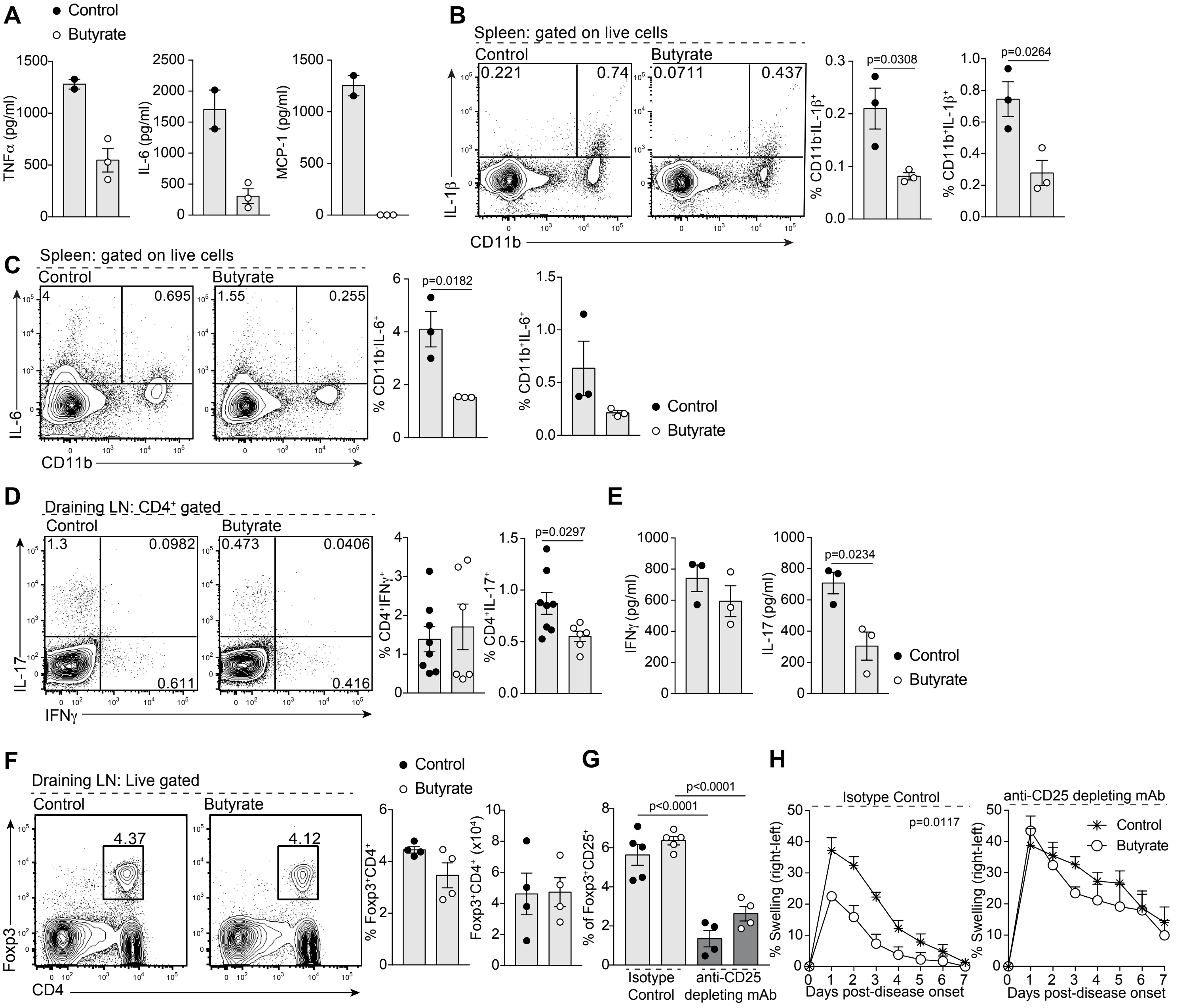

Supplementary Figure 3. Suppression of arthritis by butyrate-supplementation is associated with a reduction in pro-arthritogenic stimuli (Related to Figure 2). (A) Production of TNFα, IL-6, MCP-1 by total lymphocytes as measured by cytokine bead array (cumulative n=5 per group, one representative experiment of two experiments is shown). (B) Representative plots (left) and bar chart (right) showing the percentage of IL-1β$^+$CD11b$^-$ and IL-1β$^+$CD11b$^+$ in the spleen at day 7 post-disease onset. (C) Representative plots (left) and bar chart (right) showing the percentage of IL-6$^+$CD11b$^-$ and IL-6$^+$CD11b$^+$ and in the spleen at day 7 post-disease onset (cumulative n=8 per group, one representative experiment of two experiments is shown). (D) Representative plots (left) and bar chart (right) showing the percentage and absolute number of CD4$^+$IL-17$^+$ cells and CD4$^+$IFNγ$^+$ cells in the draining LN at day 7 post-disease onset (cumulative n=10 per group, one representative experiment of three experiments is shown). (E) Production of IL-17 and IFNγ by total lymphocytes as measured by cytokine bead array (cumulative n=5 per group, one of two repre-sentative experiments is shown). (F) Representative plots (left) and bar chart (right) showing the percentage and absolute number of CD4$^+$Foxp3$^+$ cells in the draining LN at day 7 post-disease onset (cumulative n=10 per group, one representative experiment of three experiments is shown). (G) Frequency of CD4$^+$CD25$^+$Foxp3$^+$ in the spleen of control and butyrate-supplemented mice treated with anti-CD25 depleting mAb compared to isotype control (control mice treated with anti-CD25 depleting mAb compared to isotype control: n=5 per group; butyrate-supplemented mice treated with anti-CD25 depleting mAb compared to isotype control: n=4 per group (cumulative data are shown). (H) Mean clinical score of control and butyrate-supplemented anti-CD25 depleting mAb-treated mice or isotype control mice; y axis shows percentage swelling in antigen-injected knee compared to control knee (cumulative data is shown). Data represent mean ± SE. (A-F, Student's t test; G, one-way ANOVA; H, two-way ANOVA).

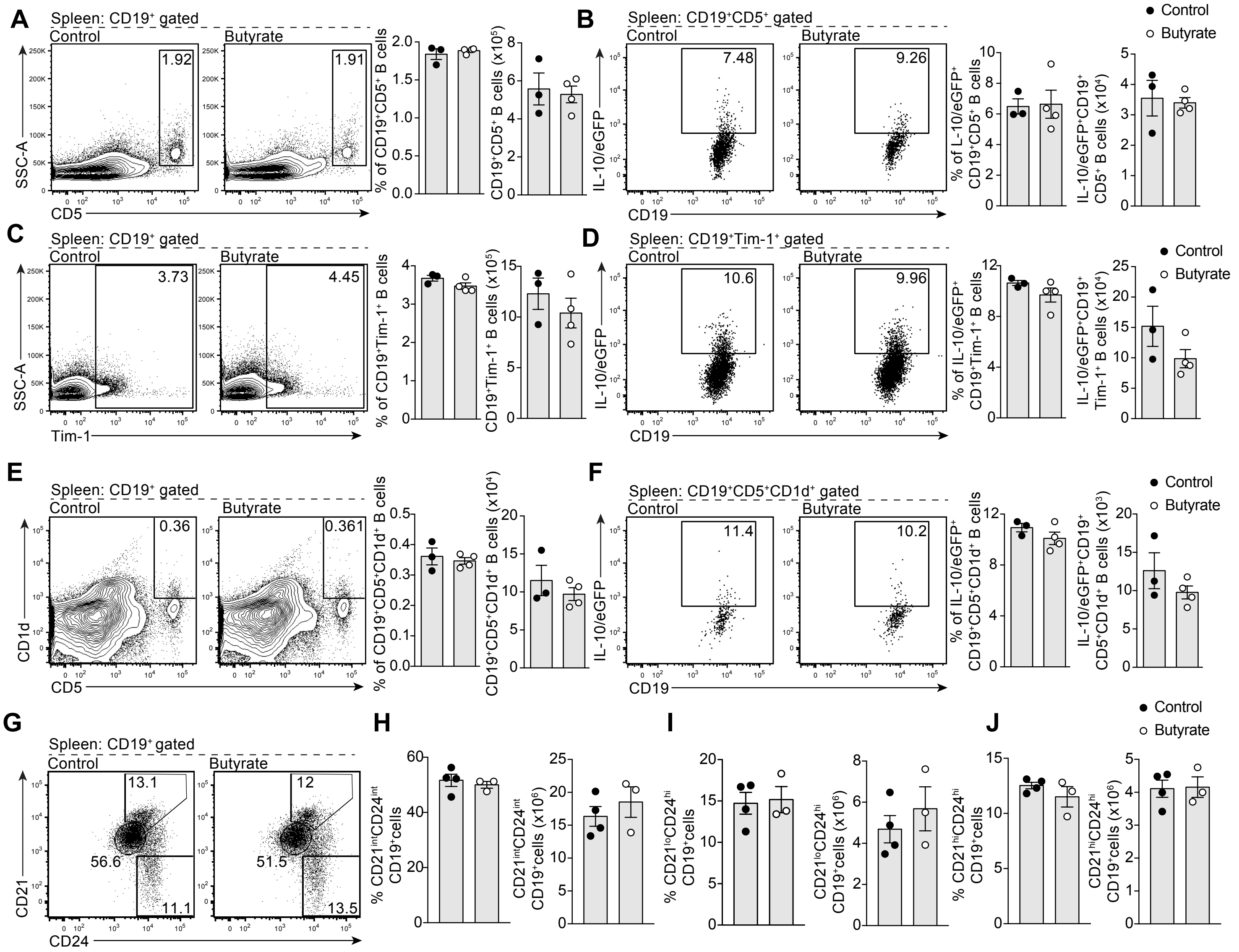

Supplementary Figure 4. Suppression of arthritis by butyrate-supplementation does not alter the frequency or number of Breg subsets or naive splenic B cell subsets (Related to Figure 2). (A) Representative plots (left) and bar chart (right) showing the percentage and number of $CD5^{+}$B cells in the spleen at day 7 post-disease onset. (B) Representative plots (left) and bar chart (right) showing the percentage and number of IL-10eGFP^{+}CD5^{+}B cells in the spleen at day 7 post-disease onset. (C) Representative plots (left) and bar chart (right) showing the percentage and number of Tim-1^{+} B cells in the spleen at day 7 post-disease onset. (D) Representative plots (left) and bar chart (right) showing the percentage and number of IL-10eGFP^{+}Tim-1^{+}B cells in the spleen at day 7 post-disease onset. (E) Representative plots (left) and bar chart (right) showing the percentage and number of CD1d^{+}CD5^{+}B cells in the spleen at day 7 post-disease onset. (F) Representative plots (left) and bar chart (right) showing the percentage and number of IL-10eGFP^{+}CD1d^{+}CD5^{+}B cells in the spleen at day 7 post-disease onset (cumulative n=8, one representative experiment of two experiments is shown). (G) Representative plots showing the percentage of CD19^{+}CD21^{int}CD24^{int}B cells, CD19^{+}CD21^{lo}CD24^{hi}B cells, and CD19^{+}CD21^{hi}CD24^{hi}B cells in the spleen at day 7 post-disease onset. Bar chart showing the percentage and number of (H) CD19^{+}CD21^{int}CD24^{int}B cells, (I) CD19^{+}CD21^{lo}CD24^{hi}B cells, and (J) CD19^{+}CD21^{hi}CD24^{hi}B cells. (Control, cumulative n=15; Butyrate cumulative n=13; one representative experiment of three experiments is shown. Data represent mean ± SE. A-J, Student's t test.

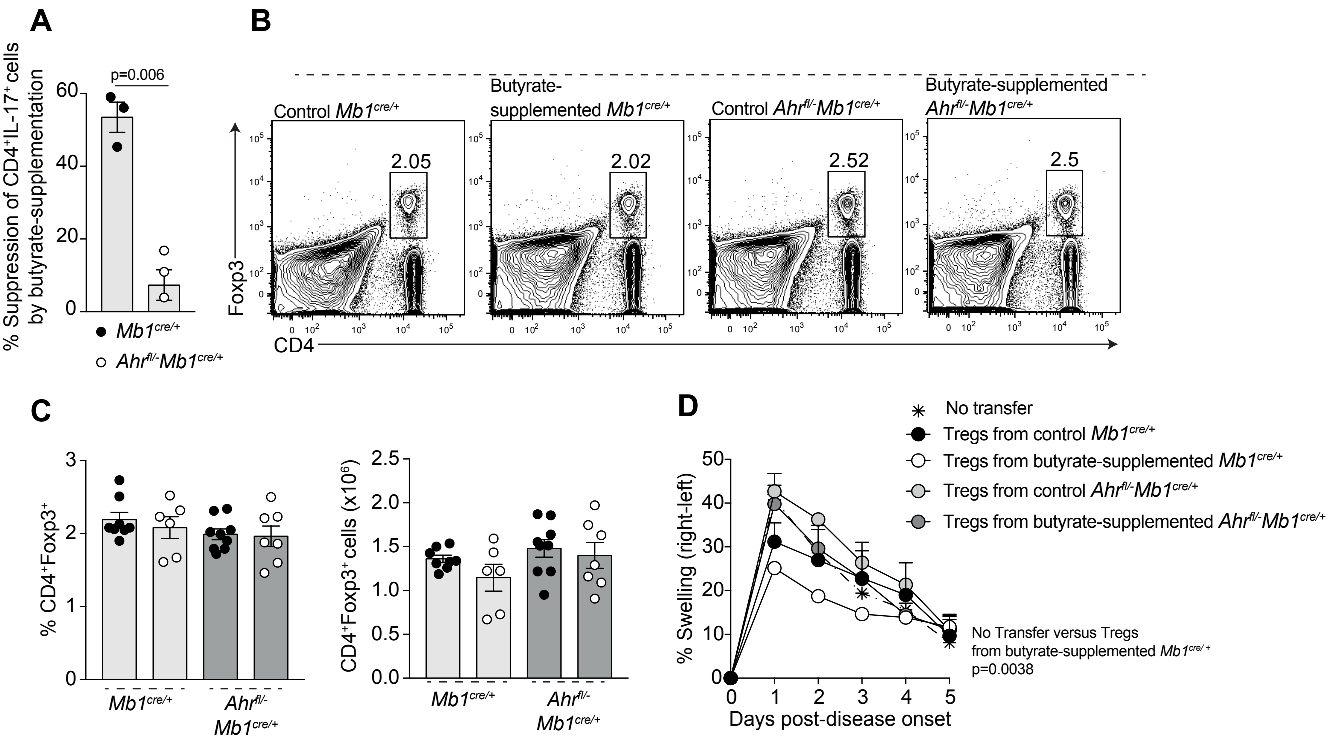

Supplementary Figure 5. Expression of AhR in B cells is fundamental for modulation of T cell function after butyrate-supplementation (Related to Figure 3). (A) Bar chart showing the suppression of $CD4^{+}IL\text{-}17^{+}$T cells in *Mb1$^{cre/+}$* or *Ahr$^{fl/-}$Mb1$^{cre/+}$* mice that received butyrate-supplementation compared to control *Mb1$^{cre/+}$* or *Ahr$^{fl/-}$Mb1$^{cre/+}$* mice (cumulative n=6 per group, one representative experiment of two experiments is shown). Representative plots (B) and bar charts (C) showing the percentage and number of $CD4^{+}Foxp3^{+}$ T cells in *Mb1$^{cre/+}$* or *Ahr$^{fl/-}$Mb1$^{cre/+}$* mice that received butyrate-supplementation compared to control *Mb1$^{cre/+}$* or *Ahr$^{fl/-}$Mb1$^{cre/+}$* mice (cumulative data are shown). (D) Mean clinical score following transfer of Tregs from control or butyrate supplemented *Mb1$^{cre/+}$* and control or butyrate supplemented *Ahr$^{fl/-}$Mb1$^{cre/+}$*, a control group that did not receive transfer; y axis shows percentage swelling in antigen-injected knee compared to control knee (n=3 per group). Data represent mean ± SE. (A, students *t* test; C, one-way ANOVA; D, two-way ANOVA).

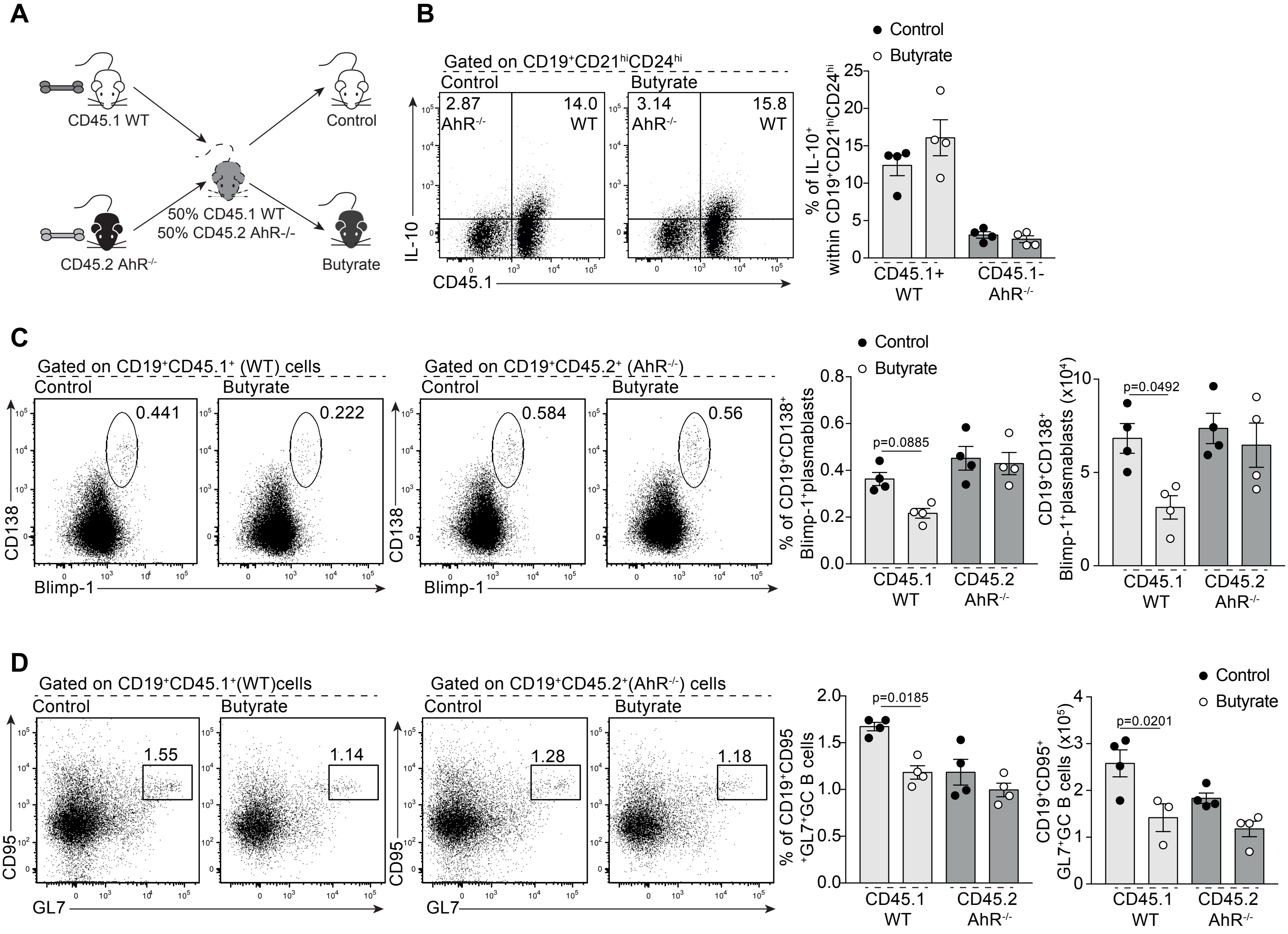

Supplementary Figure 6. Butyrate-supplementation suppresses B cell maturation through activation of AhR (Related to Figure 3). (A) Schematic showing experimental design for competitive congenic bone marrow chimeric experiment. (B) Representative flow cytometry plots (left) and bar charts (right) showing the frequency and number of CD45.1$^+$IL-10$^+$CD21^{hi}CD24$^+$ B cells or CD45.1$^-$IL-10$^+$CD21$^+$CD24$^+$B cells in butyrate-supplemented or control chimeric mice (cumulative n=5 per group). (C) Representative flow cytometry plots (left) and bar charts showing the frequency and number of plasmablasts within CD45.1$^+$WT or CD45.2$^+$ AhR$^{-/-}$ derived cells in butyrate-supplemented or control chimeric mice (cumulative n=5 per group). (D) Representative flow cytometry plots (left) and bar charts (right) showing the frequency and number of GC B cells within CD45.1$^+$ WT or CD45.2$^+$ AhR$^{-/-}$ derived cells in butyrate-supplemented or control chimeric mice (cumulative n=5 per group). Data represent mean ± SE. (B-D, one-way ANOVA).

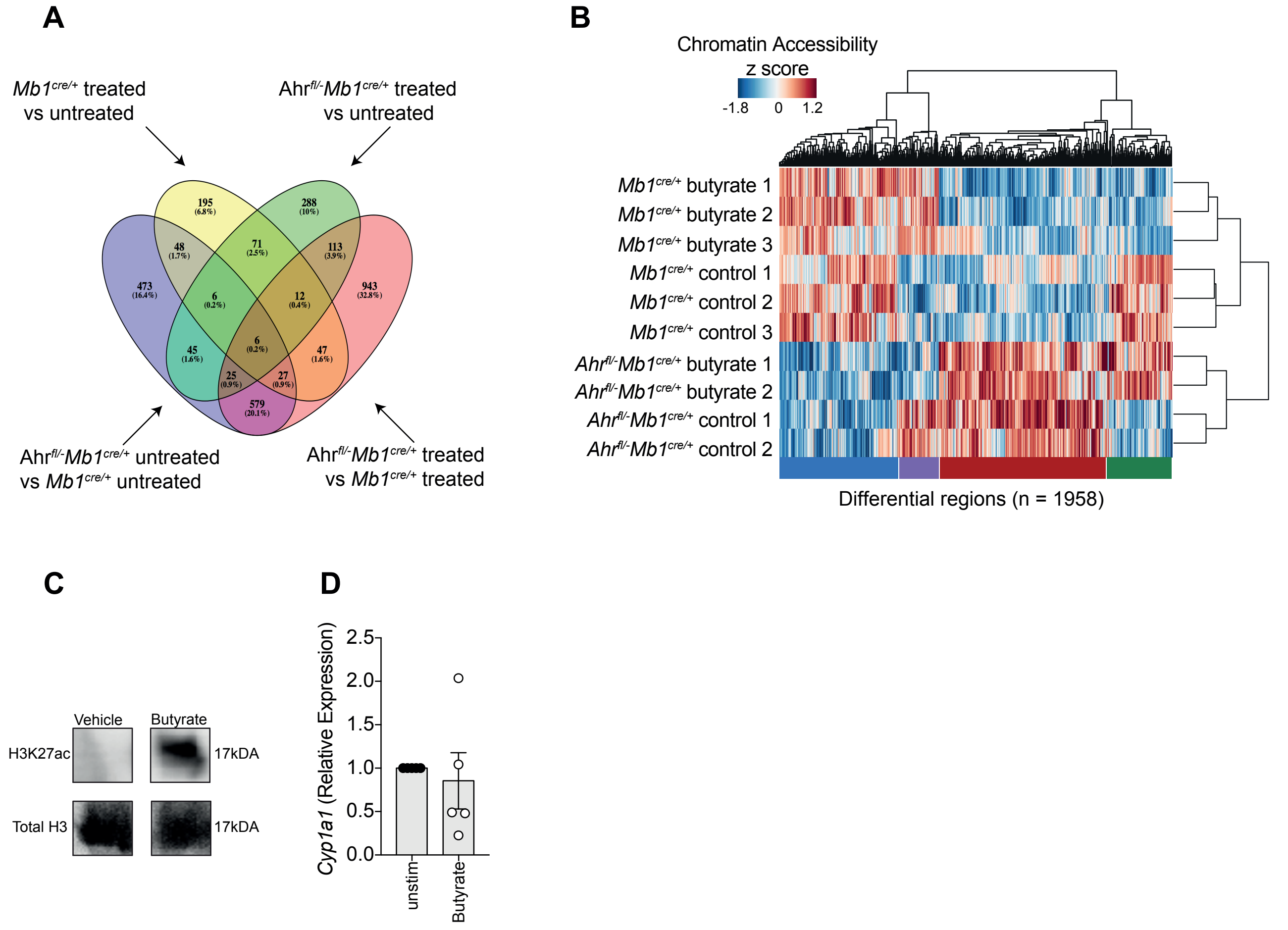

Supplementary Figure 7. Butyrate does not directly activate AhR (Related to Figure 5 and Figure 6). (A) Venn diagram indicating the number of significant ($p < 0.05$) DEG across all 4 comparisons and the number of overlapping genes between each comparison. (B) Heatmap shows differentially regulated regions of chromatin in $CD19^{+}CD21^{hi}CD24^{hi}$B cells isolated from control *Mb1*$^{cre/+}$ mice, butyrate-supplemented *Mb1*$^{cre/+}$ mice, control *Ahr*$^{fl/-}$*Mb1*$^{cre/+}$ mice, and butyrate-supplemented *Ahr*$^{fl/-}$*Mb1*$^{cre/+}$ mice as measured by ATAC-seq. (C) Total splenic B cells were isolated from WT mice and treated either with a vehicle control or 500µM butyrate for 18h and analysed for H3K27ac by Western blot. Total H3 was used as a control. The numbers indicate the size of the protein bands in kDA. (D) Bar chart shows relative expression of *Cyp1a1* following 6 hours culture with butyrate (cumulative n=5).

	Healthy control (n=20)	Rheumatoid arthritis (n=19)
**No. male/female**	4/16	4/15
**Mean age at sampling (years)**	52.6	58.5
**Treatment received within preceding 6 months of sample: None**	n/a	10.5%
**Treatment received within preceding 6 months of sample: DMARD only (%)**	n/a	26.3%
**Treatment received within preceding 6 months of sample: MTX (%)**	n/a	36.8%
**Treatment received within preceding 6 months of sample: Biological therapy (%)**	n/a	31.6%
**DAS (IQR)**	n/a	2.13 (1.61-3.06)
**CRP (μg/mL)**	n/a	1.7 (1.2-3.675)
**ESR mm/hr at time of sampling, median (IQR)**	n/a	9 (5-13)
**RF + (%)**	n/a	78.9%

Table S1: Patient demographics (Related to Figure 1).

Gene set – AhR independent butyrate regulated genes					
Adamdec1	*Casc4*	*Hip1*	*Mtfr1l*	*Pomt1*	*Tbxa2r*
Ahdc1	*Cbfa2t3*	*Hpse*	*Mtmr4*	*Ppcdc*	*Tecpr2*
Anks1	*Ddx11*	*Hsp90b1*	*Nfya*	*R3hdm1*	*Tmc4*
Ano10	*Ece1*	*Hyou1*	*Pafah2*	*Rpgrip1l*	*Tmcc3*
Ano8	*Fahd2a*	*L3mbtl3*	*Patz1*	*Sdf2l1*	*Tmem129*
Asl	*Fam173b*	*Lamc1*	*Pdia3*	*Slc16a6*	*Tnfrsf4*
Bcl2	*Fbxl5*	*Lman2l*	*Pdia6*	*Slc2a9*	*Unc119b*
Bcl9	*Fkbp2*	*Magt1*	*Piga*	*Slc37a2*	*Usp31*
Bicd2	*Flnb*	*Manf*	*Pik3r5*	*Smg7*	*Vti1a*
Calr	*Fuca1*	*Med16*	*Plod1*	*Sorbs3*	*Xbp1*
Canx	*Gprasp1*	*Mib2*	*Plxna1*	*St13*	*Znhit1*
Capn5	*Guca1b*	*Mrpl1*	*Poln*	*Tbc1d19*	

Table S2: AhR independent butyrate regulated genes (Related to Figure 4). 71 identified genes which are significantly differentially expressed after butyrate-supplementation in both *Mb1*$^{cre/+}$ and *Mb1*$^{cre/+}$*Ahr*$^{fl/-}$ mice.

**Gene Set – AhR-dependent butyrate regulated genes**					
1110065P20Rik	*Cchcr1*	*Gfod1*	*Nacc1*	*Rab26os*	*Top3a*
1700048O20Rik	*Cd180*	*Haghl*	*Nek1*	*Rala*	*Tpst1*
1810014B01Rik	*Cep104*	*Haus2*	*Nfe2l2*	*Recql*	*Trmt10b*
1810024B03Rik	*Cep162*	*Hdac11*	*Nfkbiz*	*Rnase12*	*Trmt2b*
2010111I01Rik	*Cep78*	*Hist1h4d*	*Noa1*	*Rpl12*	*Tsc22d1*
2500004C02Rik	*Cers4*	*Ift74*	*Nod1*	*Rpl37*	*Ttc13*
3110009E18Rik	*Cgrrf1*	*Ints3*	*Nt5c2*	*Rpn1*	*Unc119*
4632415L05Rik	*Chid1*	*Ints9*	*Oplah*	*Rps19-ps3*	*Urb1*
4833418N02Rik	*Creld1*	*Ipmk*	*Ovgp1*	*Rundc3b*	*Utp4*
4930402H24Rik	*Creld2*	*Ipp*	*Oxsm*	*Sel1l3*	*Vmac*
6030419C18Rik	*Cwc27*	*Itga10*	*P2rx7*	*Selenoi*	*Vps37b*
A430033K04Rik	*Cyp4v3*	*Kcnk13*	*Pacs2*	*Slamf1*	*Wdr62*
A530072M11Rik	*Dbp*	*Kctd1*	*Pcgf3*	*Slc12a3*	*Xpnpep3*
Adam15	*Dcxr*	*Kctd17*	*Pde6d*	*Slc12a5*	*Xrn2*
AI504432	*Dedd2*	*Kifc5b*	*Pde8a*	*Slc17a9*	*Zc3h12b*
Aldh1l2	*Dip2a*	*Ldhd*	*Pfkfb1*	*Slc25a1*	*Zdhhc20*
Arid3b	*Dirc2*	*Lrp11*	*Pfkfb4*	*Slc2a8*	*Zdhhc7*
Arl6ip4	*Dnajb11*	*Lrpap1*	*Pgp*	*Slc30a4*	*Zfp112*
Asphd1	*Dqx1*	*Lta*	*Pi4k2a*	*Spast*	*Zfp128*
Atg10	*Dscr3*	*Mapre3*	*Pik3ip1*	*Spata24*	*Zfp229*
Atp9a	*Eif2b4*	*Marf1*	*Plk2*	*Ssbp2*	*Zfp236*
Atrn	*Emsy*	*Mccc2*	*Ppard*	*St3gal1*	*Zfp280c*
Atxn2	*Ergic1*	*Med26*	*Ppm1d*	*Stxbp4*	*Zfp292*
Baiap2	*Evi5*	*Mettl22*	*Ppm1l*	*Susd2*	*Zfp39*
BC051142	*Fam120c*	*Mettl23*	*Ppp1r35*	*Suv39h2*	*Zfp438*
Begain	*Fam241a*	*Mfsd1*	*Ppp2r1b*	*Taf3*	*Zfp446*
Bloc1s4	*Fbf1*	*Mfsd2a*	*Praf2*	*Tbl1x*	*Zfp568*
Bmt2	*Fchsd1*	*Mlkl*	*Prelid3b*	*Tfcp2*	*Zfp729a*
Btbd18	*Flot1*	*Mrpl33*	*Prkar2a*	*Timd2*	*Zfp943*
Camk2a	*Fndc10*	*Mterf3*	*Prmt6*	*Tmem165*	*Zfyve21*
Cars	*Gabbr1*	*Mturn*	*Psph*	*Tmem71*	*Znrf1*
Ccdc85b	*Galnt7*	*Mzt2*	*Ptger1*	*Tomm6os*	*Zscan22*
	Gfm2		*Pycr1*		*Zscan26*

Table S3: AhR-dependent butyrate regulated genes (Related to Figure 4). 195 identified genes which were significantly differentially expressed after butyrate-supplementation in *Mb1*$^{cre/+}$ mice, once baseline transcriptional changes between control *Mb1*$^{cre/+}$ mice versus *Mb1*$^{cre/+}$*Ahr*$^{fl/-}$ mice had been removed.
